# Identification of putative regulatory regions and transcription factors associated with intramuscular fat content traits

**DOI:** 10.1186/s12864-018-4871-y

**Published:** 2018-06-27

**Authors:** Aline S. M. Cesar, Luciana C. A. Regitano, James M. Reecy, Mirele D. Poleti, Priscila S. N. Oliveira, Gabriella B. de Oliveira, Gabriel C. M. Moreira, Maurício A. Mudadu, Polyana C. Tizioto, James E. Koltes, Elyn Fritz-Waters, Luke Kramer, Dorian Garrick, Hamid Beiki, Ludwig Geistlinger, Gerson B. Mourão, Adhemar Zerlotini, Luiz L. Coutinho

**Affiliations:** 1 Department of Animal Science, University of São Paulo, Piracicaba, SP 13418-900 Brazil; 2 Department of Animal Science, Iowa State University, Ames, IA 50011 USA; 3 Embrapa Pecuária Sudeste, São Carlos, SP 13560-970 Brazil; 4 Embrapa Informática Agropecuária, Campinas, SP 13083-886 Brazil; 5 School of Agriculture, Massey University, Ruakura, Hamilton, New Zealand

**Keywords:** eQTL, Metabolic diseases, Fatty acids, Mammals, Gene expression

## Abstract

**Background:**

Integration of high throughput DNA genotyping and RNA-sequencing data allows for the identification of genomic regions that control gene expression, known as expression quantitative trait loci (eQTL), on a whole genome scale. Intramuscular fat (IMF) content and carcass composition play important roles in metabolic and physiological processes in mammals because they influence insulin sensitivity and consequently prevalence of metabolic diseases such as obesity and type 2 diabetes. However, limited information is available on the genetic variants and mechanisms associated with IMF deposition in mammals. Thus, our hypothesis was that eQTL analyses could identify putative regulatory regions and transcription factors (TFs) associated with intramuscular fat (IMF) content traits.

**Results:**

We performed an integrative eQTL study in skeletal muscle to identify putative regulatory regions and factors associated with intramuscular fat content traits. Data obtained from skeletal muscle samples of 192 animals was used for association analysis between 461,466 SNPs and the transcription level of 11,808 genes. This yielded 1268 *cis-* and 10,334 *trans*-eQTLs, among which we identified nine hotspot regions that each affected the expression of > 119 genes. These putative regulatory regions overlapped with previously identified QTLs for IMF content. Three of the hotspots respectively harbored the transcription factors *USF1*, *EGR4* and *RUNX1T1*, which are known to play important roles in lipid metabolism. From co-expression network analysis, we further identified modules significantly correlated with IMF content and associated with relevant processes such as fatty acid metabolism, carbohydrate metabolism and lipid metabolism.

**Conclusion:**

This study provides novel insights into the link between genotype and IMF content as evident from the expression level. It thereby identifies genomic regions of particular importance and associated regulatory factors. These new findings provide new knowledge about the biological processes associated with genetic variants and mechanisms associated with IMF deposition in mammals.

**Electronic supplementary material:**

The online version of this article (10.1186/s12864-018-4871-y) contains supplementary material, which is available to authorized users.

## Background

High-throughput genotyping and gene expression analysis combined with increased computational capacity and robust statistical methods allows for the identification of expression quantitative trait loci (eQTL). This approach can identify genomic regions that are associated with gene expression level [1–3] and can help to elucidate molecular mechanisms whereby genomic variants exert their effects on phenotypes or disease incidence [4, 5].

Adipose tissue is the largest endocrine organ in the body [6]. Within skeletal muscle, adipose deposition can occur within (intramyocellular) and external to (extramyocellular) skeletal muscle fibers. Lipid stored in skeletal muscle plays important roles in metabolic processes such as energy homeostasis, expression and secretion of hormones and proinflammatory cytokines, and in signaling pathways [7].

In humans, excessive fat deposition in skeletal muscle has been associated with metabolic diseases such as obesity, diabetes and coronary heart disease [8]. In contrast, in bovine, swine, and sheep, intramuscular fat (IMF) is positively associated with meat quality and consumer satisfaction [9], and can affect the final product price. Furthermore, the quantity and the fatty acid profile of the IMF present in edible red meat have both been positively and negatively associated with human health [10–12].

It is important to gain additional knowledge about the biological processes associated with IMF deposition and composition because of the important role of IMF deposition in areas such as human health and meat quality. Previously, Uemoto et al. [13], Ishii et al. [14], and our group have reported the identification of several quantitative trait loci (QTL) and putative candidate genes associated with IMF using high density SNP chip data [15]. In addition, we reported the identification of differentially expressed genes and putative candidate regulatory genes associated with IMF from RNA sequencing data (RNA-Seq) [16]. Furthermore, we have reported the identification of differentially expressed genes and gene networks associated with fatty acid composition in skeletal muscle [17]. However, so far there is no information on how genetic variation can influence gene expression and phenotypic variation associated with IMF content traits and composition [18–20]. Additional information can be gleaned by integrating the data of gene expression and whole genome association. Thus, the aim of this study was to perform eQTL analysis to identify putative regulatory regions and transcription factors (TFs) associated with intramuscular fat (IMF) content traits.

## Results

### Phenotypic, genotypic and RNA-Seq data

After the application of quality control filters, 461,466 markers and 11,808 genes from 192 animals were used in these analyses. The descriptive statistic of all phenotypes related to fat deposition and composition are shown in Additional file 1. The variance components and genomic heritability of these phenotypes for the whole population of this study have been estimated and previously reported by Cesar et al. [15] and Tizioto et al. [16]. The heritability of these phenotypes ranged from 0.09 to 0.46 (Table 1).

**Table 1:** Posterior means of variance components for IMF deposition and composition in Nellore by Bayes B

Trait	Genetic variance	Residual variance	Total variance	Genomic heritability^a^
BFT (mm)^b^	0.78	2.87	3.65	0.21
IMF (%)	0.16	0.48	0.64	0.25
Myristic (%)	0.06	0.23	0.29	0.2
Myristoleic (%)	0.03	0.02	0.05	0.25
Palmitic (%)	2.42	5.47	7.89	0.31
Palmitoleic (%)	0.1	0.3	0.4	0.24
Stearic (%)	1.23	5.28	6.51	0.19
Oleic (%)	6.08	7.11	13.19	0.46
Linoleic (%)	0.00007	0.0003	0.00037	0.19
CLAc9t11 (%)	0.0003	0.002	0.0023	0.12
SFA (%)	1.36	15.54	16.9	0.08
MUFA (%)	1.67	15.18	16.85	0.1
PUFA (%)	0.09	0.54	0.63	0.14
Sum_n-3 (%)	0.004	0.013	0.017	0.25
Sum_n-6 (%)	0.0005	0.003	0.0035	0.15

^a^Cesar et al. [15]

^b^Tizioto et al. [16]

### Identification of eQTLs and the genomic location of variants

**Fig. 1:**
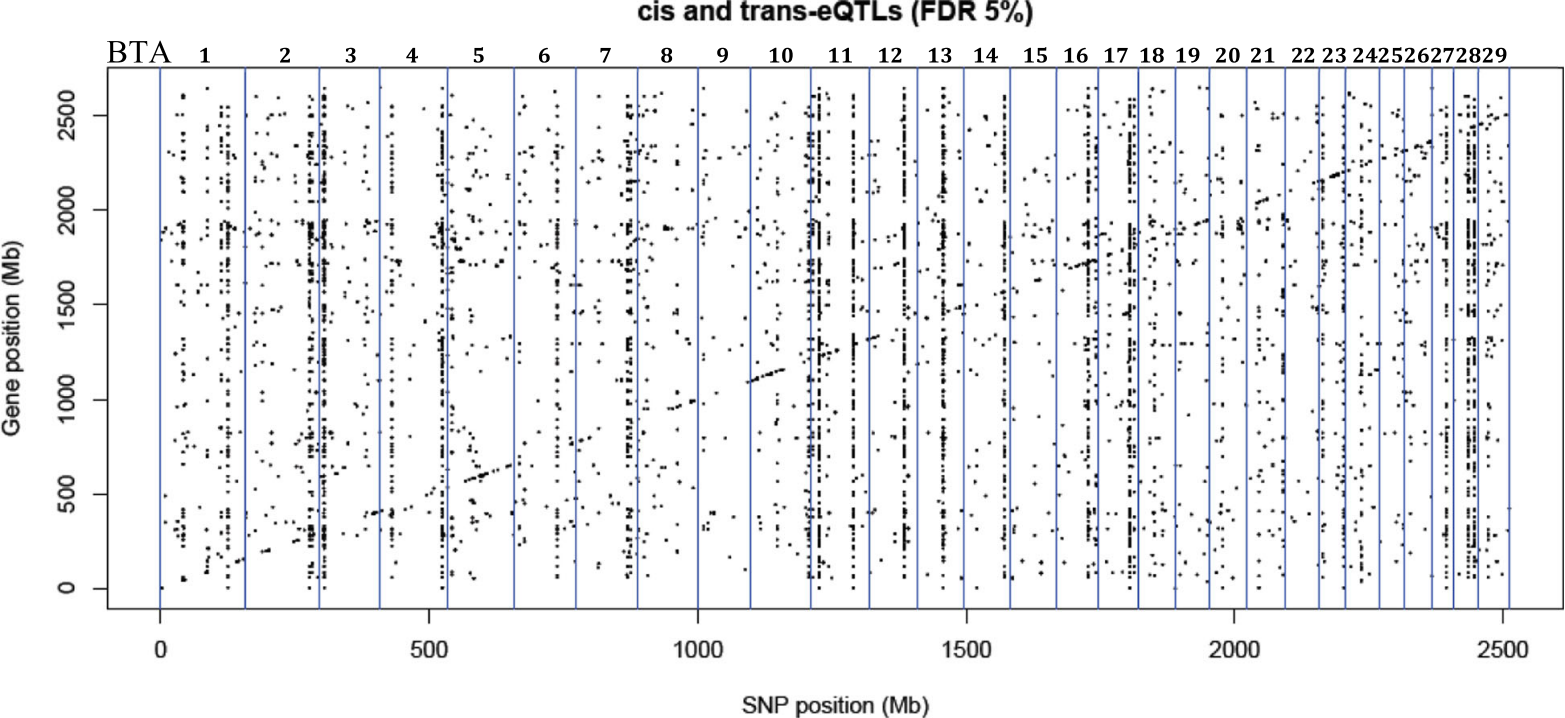
A scatter plot of gene location (Mb) versus significant eQTL position (Mb). The vertical blue lines denote individual chromosomes. Points distributed diagonally indicate cis-eQTLs. Points distributed vertically indicate trans-eQTLs

In total, 10,334 trans-eQTLs (variants that were located more than 1 Mb from an associated gene) and 1268 cis-eQTL (variants located within 1 Mb of the associated gene) were identified herein (5% false discovery rate, FDR) (see Additional files 2 and 3) by MatrixeQTL R package. Figure 1 shows a scatter plot of gene location (Mb) relative to its significant corresponding eQTL position (Mb).

The cis-eQTLs were located in intronic (46%), intergenic (30%), upstream (11%), downstream (10%), and 3’ UTR (1%) regions (see Additional file 4A). The trans-eQTLs were located in intergenic (61%), intronic (30%), upstream (4%) and downstream (4%) regions (see Additional file 4B). Analysis of the associated SNPs located in coding regions (less than 1 %) revealed that 66 and 34% of cis-eQTLs (see Additional file 5) and 52 and 48% of trans-eQTLs (see Additional file 6) were either synonymous (not causing a change in the protein sequence) or missense (causing a change in the protein sequence) variants, respectively.

### Overlap test between eQTLs and QTLs

**Fig. 2:**
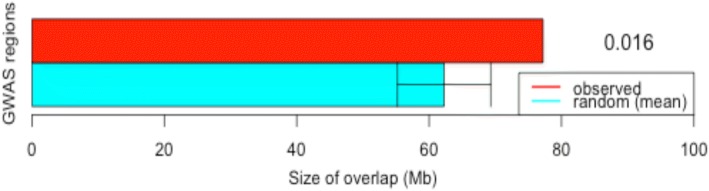
QTLs of the Cattle QTLdb associated with deposition and composition of intramuscular fat that overlap with eQTLs regions. Red bars correspond to the mean overlap size (in Mb) of the eQTL regions that was observed for eQTL regions for respective trait (y-axis), while the cyan bars indicate the mean overlap size (in Mb) estimated after 1000× random resamplings (x-axis). The error bars indicate the standard deviation, while permutation *p-*values are listed on the right

Previous studies observed that many QTL are not located within coding regions [17, 21, 22], which might indicate that the causative mutations control gene expression level rather than altering gene function as would a non-synonymous exonic mutation. To determine if the eQTLs identified in this study overlap (see Additional file 7) with annotated QTL in Cattle QTLdb [23], a permutation test (*p*-value < 0.05) was performed using the regioneR package [23]. We observed that 2920 eQTLs (24%) overlapped QTLs in the Cattle QTLdb [23] with a total basewise overlap of 927.2 Mb, which corresponds to 61% of the genomic regions covered by eQTLs. In 47.4% of the cases, the eQTLs completely overlapped with QTL regions from Cattle QTLdb. A permutation test was conducted to evaluate if eQTL overlapped with each of the general QTL classes reported in Cattle QTLdb. In all cases, eQTL overlapped QTL in all general QTL classes to a significantly larger extent than expected by chance. The QTLs (see Additional file 8) associated with deposition and composition of intramuscular fat previously reported by our group [15] (Fig. 2) as well as QTLs associated with beef production and carcass and beef quality also had an overlap with eQTLs significantly larger than expected by chance.

### Identification of eQTL hotspots, their functional annotation and effect on IMF content traits

**Fig. 3:**
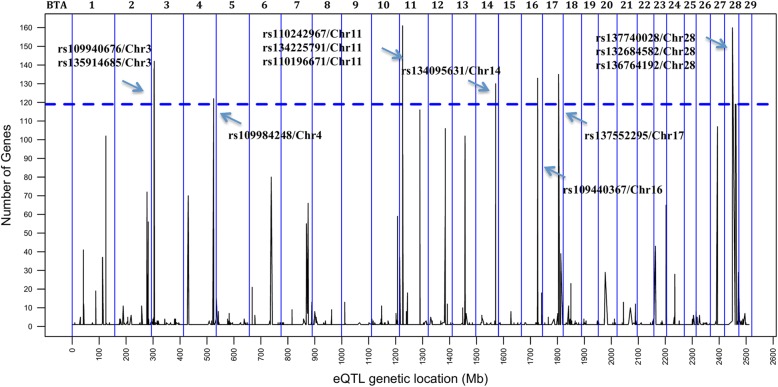
The eQTL hotspot regions across the Nellore genome identified in skeletal muscle. The blue dotted line shows the threshold (≥ 119) of number of genes associated with a single marker. The vertical blue lines denote individual chromosomes

Mutations that influence the expression of several genes (eQTL hotspots) can modulate metabolic pathways and as a result can cause changes in phenotype [25]. Based on this definition of a hotspot, a permutation test was performed (*p* < 0.05), which indicated that a given variant needed to be associated with the expression level of at least 119 genes. We identified 12 significant hotspots on BTA3, BTA4, BTA11, BTA14, BTA16, BTA17 and BTA28 (Fig. 3). Because some of these eQTL hotspots were located near each other, we examined the linkage disequilibrium (LD) among them and refined the eQTL hotspot regions to nine, located on BTA3 (8 Mb), BTA4 (108 Mb), BTA11 (11 Mb), BTA14 (73 Mb), BTA16 (59 Mb), BTA17 (55 Mb) and BTA28 (20 Mb, 32 Mb). The LD observed for hotspots on BTA11, BTA28, and also between each pair of the hotspots are shown in Additional files 9 and 10, respectively. The association test to verify the effect of all eQTL hotspots on the IMF content traits were performed using an ANOVA test (Table 2) (see Additional file 11) with correction for multiple tests by calculating the false discovery rate (FDR 5%).

**Table 2:** Association test between the three eQTL hotspots that harbor TF in their region, and IMF content traits

eQTL hotspots	rs135914685- hotspot 1(*USF1*)		rs110242967 - hotspot 2 (*EGR4*)		rs134095631 - hotspot 3 (*RUNX1T1*)	
Traits	*p*-value	FDR^1^	*p*-value	FDR	*p*-value	FDR
BFT^a^	1.89E-06	7.47E-06^i^	2.77E-06	8.30E-06^i^	8.30E-06	2.07E-05^i^
IMF^b^(%)	3.55E-02	6.61E-02	7.61E-02	8.15E-02	3.24E-02	3.74E-02^i^
Myristic (%)	5.45E-04	2.30E-03^i^	1.51E-05	2.84E-05^i^	1.40E-05	2.63E-05^i^
Myristoleic (%)	2.73E-05	1.14E-04^i^	3.61E-04	5.41E-04^i^	5.65E-04	7.71E-04^i^
Palmitic (%)	5.37E-07	7.47E-06^i^	5.34E-07	2.00E-06^i^	5.55E-07	2.08E-06^i^
Palmitoleic (%)	1.21E-05	5.45E-05^i^	9.73E-06	2.09E-05^i^	1.20E-05	2.58E-05^i^
Stearic (%)	1.38E-01	1.89E-01	1.21E-01	1.21E-01	1.61E-01	1.61E-01
Oleic (%)	2.04E-14	5.17E-13^i^	2.11E-14	1.06E-13^i^	1.76E-14	8.82E-14^i^
Linoleic (%)	2.74E-02	4.27E-02^i^	3.65E-02	4.21E-02^i^	4.22E-02	4.53E-02^i^
CLAc9t11^c^(%)	2.20E-16	3.30E-15^i^	2.20E-16	3.30E-15^i^	2.20E-16	3.30E-15^i^
SFA^d^(%)	4.78E-06	3.75E-05^i^	4.62E-06	1.16E-05^i^	4.09E-06	1.23E-05^i^
MUFA^e^(%)	1.72E-05	8.81E-05^i^	1.66E-04	2.76E-04^i^	1.41E-04	2.35E-04^i^
PUFA^f^(%)	3.09E-04	1.26E-03^i^	4.03E-04	5.49E-04^i^	4.03E-04	6.04E-04^i^
Sum_n3^g^(%)	2.20E-16	3.30E-15^i^	2.20E-16	1.65E-15^i^	2.20E-16	1.65E-15^i^
Sum_n6^h^(%)	1.24E-02	3.80E-02^i^	1.10E-02	1.38E-02^i^	9.68E-03	1.21E-02^i^

^a^Backfat thickness

^b^intramuscular fat

^c^conjugated linoleic acid cis9 trans11

^d^saturated fatty acid

^e^monounsaturated fatty acid

^f^polyunsaturated fatty acid

^g^sum of omega-3, and

^h^sum of omega-6

^i^Significant FDR 5%

^1^FDR – Adjusted *p*-value using the false discovery rate method by Benjamini and Hochberg 1995

Among the 244 genes identified within the hotspots (4 Mb windows), there were four transcription factors: *EGR4*, *RUNX1T1*, *NR1I3* and *USF1*; four miRNAs: bta-mir-2294, bta-mir-1584, bta-mir-2322, bta-mir-584-3; two nuclear receptors: *NCOR2* and *NRBF2*; three small nuclear RNAs: *SNORA70*e, *SNORA19* and *SNORA9*; and several important genes associated with cellular signaling and translation initiation such as: *CACYBP*, *CASP2*, *DIABLO*, *FSBP*, *GEM*, *RGS5* and *EIF2B1*.

In order to better understand the underlying biological processes associated with the hotspot eQTLs, PANTHER [26] was used to perform functional enrichment analysis, which uses the over-represented test (nominal *p*-value ≤0.05, before correction for multiple tests of pathways). In this analysis, the list of all genes expressed in skeletal muscle (data from this study) was used as the background or reference gene list. Additional enrichment analysis was conducted with the list of all annotated genes harbored within 4 Mb (2 Mb for each side) of cis (243 genes), trans (1453 genes) and hotspot (133 genes) eQTL regions (see Additional file 12A, B and C, respectively). Based on the results from functional enrichment, we suggest that the genes harbored within the eQTL regions are likely to be involved in modulation of transcription, translation and catalytic activity based on the GO terms associated with the genes in eQTL regions (see Additional file 12).

### Transcription factor binding sites

**Fig. 4:**
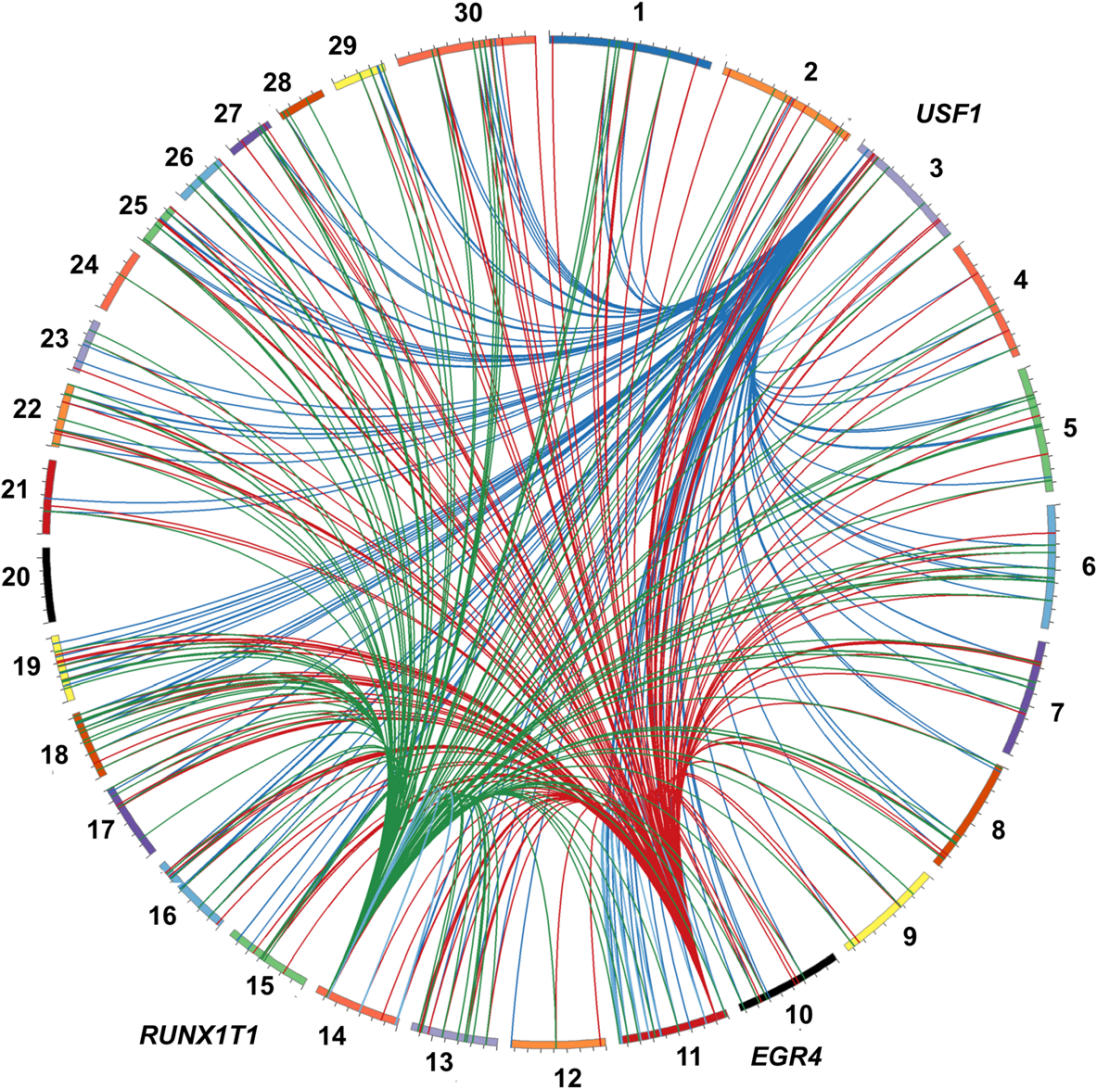
Circos plot of eQTL hotspot regions that harbored the transcription factors (TF) *EGR4* (rs110242967), *RUNX1T1* (rs134095631) and *USF1* (rs135914685). Colored boxes represent each chromosome while the colored lines show the association between the regions that harbor the TFs and the genes that were associated with these hotspot regions. *USF1* region connections are shown in blue: *EGR4* region connections are shown in red: *RUNX1T1* region connections are shown in green

Transcription factors modulate gene expression by binding to specific DNA regions [27]. To confirm that the identified transcription factors found in the eQTL hotspot regions (*EGR4*, *RUNX1T1* and *USF1)* were modulating the differentially expressed genes, we determined the presence of TFBS in the promoter regions of the affected genes by LASAGNA (Length-Aware Site Alignment Guided by Nucleotide Association, algorithm) Search 2.0 [28]. Based on LASAGNA results, about 98% of the promoters of genes in regions associated with a hotspot region had a TFBS (*p*-value ≤0.05) for the respective TF (see Additional files 13, 14 and 15, respectively). The Circos plot in Fig. 4 shows the links between the eQTL hotspots that contained the transcription factors, *EGR4*, *USF1*, and *RUNX1T1*, and their associated target genes. The over-represented TFBS motifs of *EGR4*, *RUNX1T1* and *USF1* observed in the promoters of genes within hotspot eQTL regions are shown in Additional file 16.

### Co-expression networks and correlation with traits

We used weighted correlation network analysis (WGCNA) to further explore how the three eQTL hotspot regions that harbor the TFs could affect gene expression and phenotype. For that, in each hot spot region, we selected the SNP with lowest *p* value to compare alternative genotypes for the hotspot. Unfortunately, we could not compare alternative homozygous genotypes because there were only two or three BB animals. So, the comparison was made between the homozygous AA vs heterozygous AB genotypes. This approach allowed us to identify the pattern of co-expressed genes assigned to various co-expression modules for both AA and AB genotypes and correlate them to the different traits related to IMF content traits. This correlation can represent the set of co-expressed genes that are associated with biological processes involved in lipid metabolic process in skeletal muscle.

**Fig. 5:**
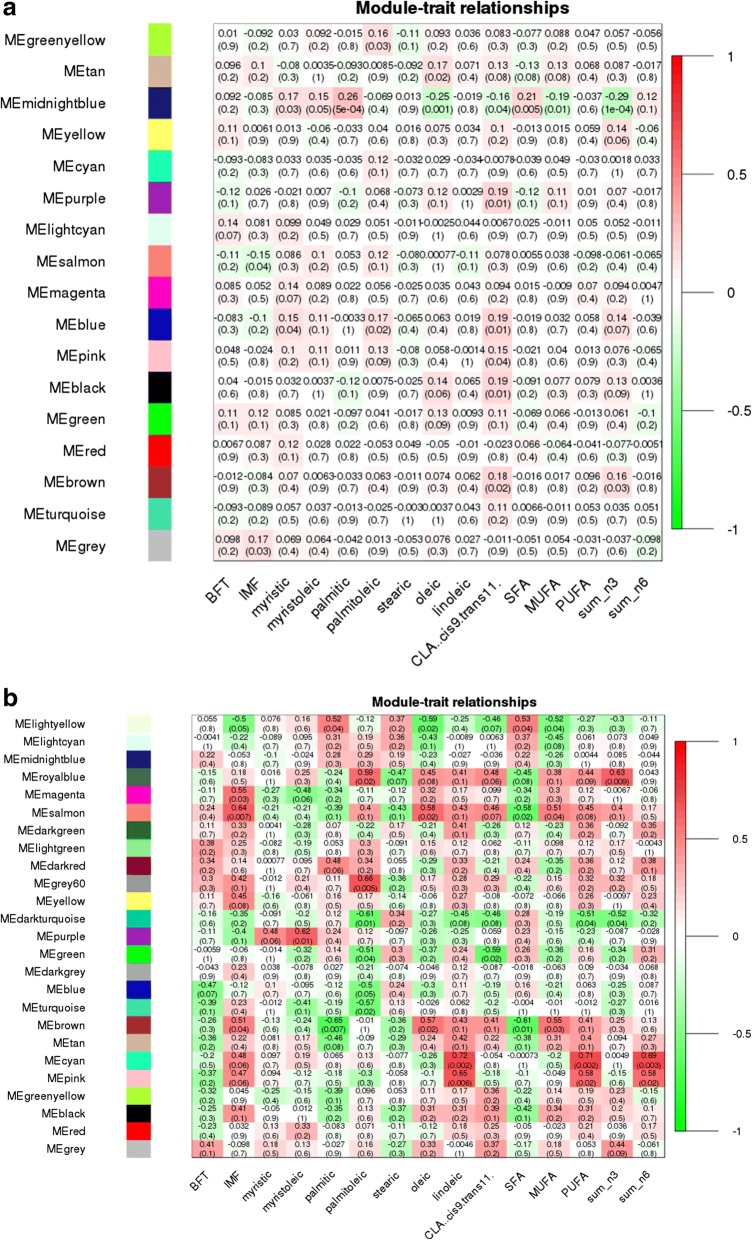
**a** Correlation between network module eigengene (ME) values and traits. Colors to the left represent the 17 gene expression modules identified from AA genotype (rs135914685) individuals. **b** Correlations between network module eigengene values and traits. Colors to the left represent the 25 modules in the AB genotype (rs135914685) network. For each module, the heatmap shows ME correlations to traits. Numbers in each cell report the correlation coefficients and student asymptotic p-value (parentheses) for significant ME-trait relationships. The scale bar, on the right, indicates the range of correlations from positive (red, 1) to negative (green, − 1)

**Fig. 6:**
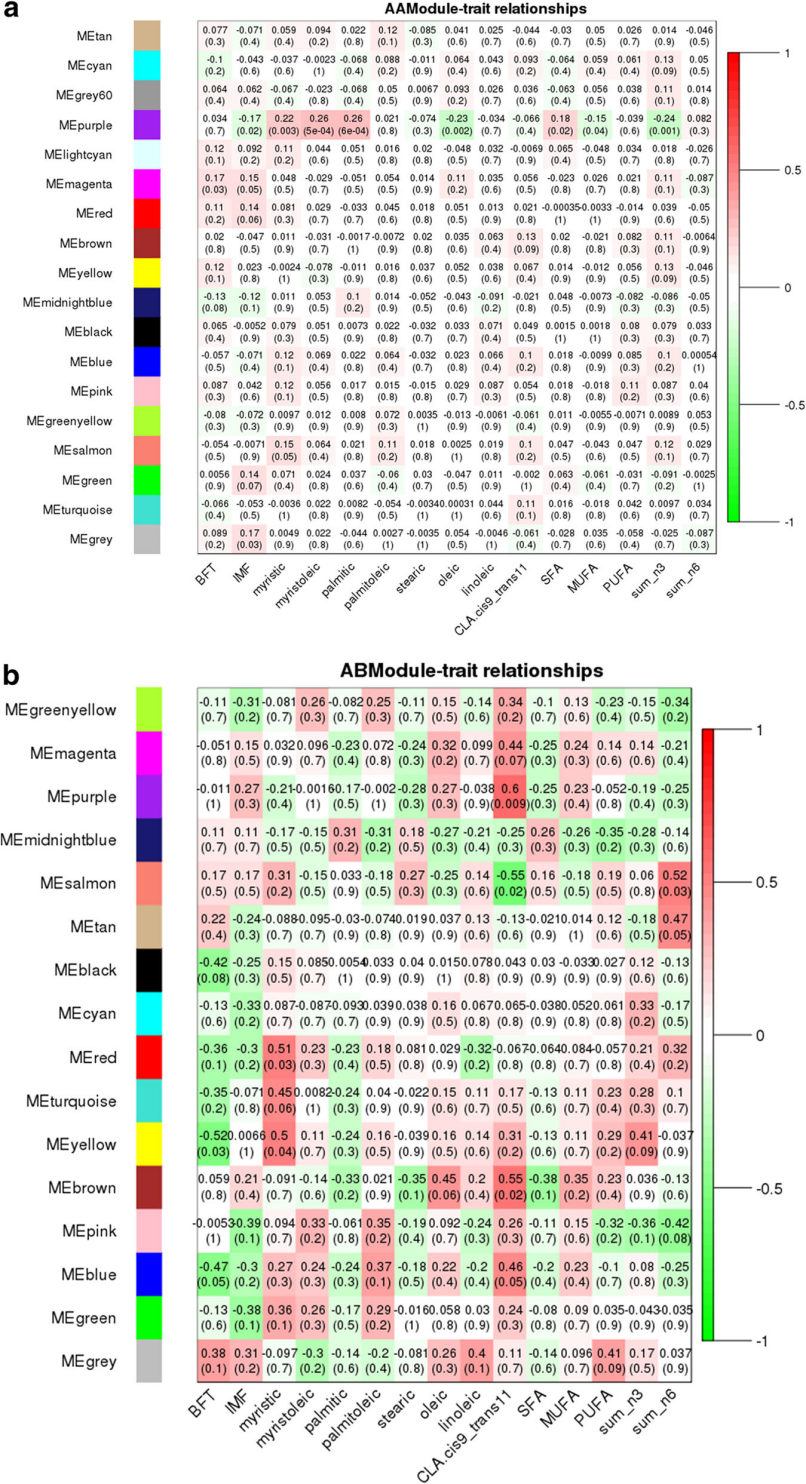
**a** Correlation between network module eigengene values and traits. Colors to the left represent the 18 modules in the AA genotype (rs110242967) network. **b** Correlation between network module eigengene (ME) values and traits. Colors to the left represent the 16 modules in the AB genotype (rs110242967) network. For each module, the heatmap shows ME correlations to traits. Numbers in each cell report the correlation coefficients and student asymptotic p-value (parentheses) for significant ME-trait relationships. The scale bar, on the right, indicates the range of correlations from positive (red, 1) to negative (green, − 1)

**Fig. 7:**
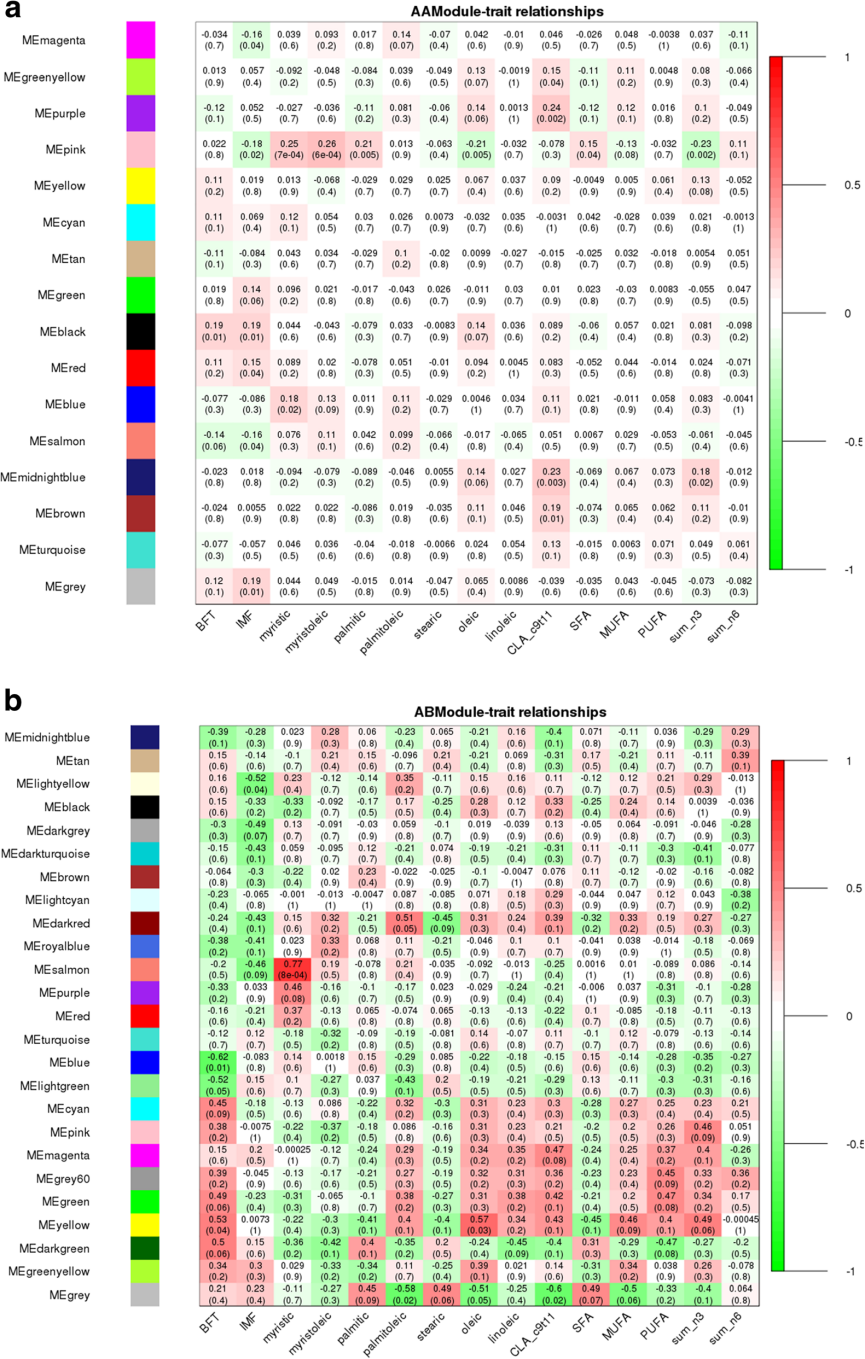
**a** Correlation between network module eigengene values and traits. Colors to the left represent the 16 modules in the AA genotype (rs134095631) network. **b** Correlation between network module eigengene (ME) values and traits. Colors to the left represent the 25 modules in the AB genotype (rs134095631) network. For each module, the heatmap shows ME correlations to traits. Numbers in each cell report the correlation coefficients and student asymptotic p-value (parentheses) for significant ME-trait relationships. The scale bar, on the right, indicates the range of correlations from positive (red, 1) to negative (green, − 1)

The eQTL hotspot region that harbored the *USF1* TF (rs135914685) presented 17 and 25 modules for AA (*n* = 175) and AB (*n* = 17) genotype, respectively (Fig. 5a and b). The *EGR4* TF (rs110242967) presented 18 modules for the AA (*n* = 174) and 16 for AB (*n* = 18) genotype, respectively (Fig. 6a and b). The eQTL hotspot region that harbored the *RUNX1T1* TF (rs134095631) presented 16 and 25 modules for AA (*n* = 176) and AB (*n* = 16) genotype, respectively (Fig. 7a and b). After estimating the correlation values (r) between the modules and each trait of interest, modules that were correlated with at least three different traits were selected for functional enrichment analysis (*p*-value < 0.1). While the number of animals with a given hotspot genotype was very similar among the hotspot groups, the set of animals within a given hotspot genotype was different. It is important to point out that there is no selection for fat deposition or composition in this population.

### Hotspot 1 - rs135914685, BTA3: 8117390-10117390, *USF1*

The AA-modules named “black”, “blue”, “green”, “midnightblue”, “pink”, “purple”, “salmon”, and “tan” were correlated (*p*-value < 0.10) with at least three different traits as shown in Fig. 5a. The “midnightblue” module presented higher correlation values (greater than 0.17) and was significantly correlated (*p*-value < 0.10) with eight of the 15 phenotypes studied.

For the AB-modules the “black”, “brown”, “cyan”, “darkturquoise”, “lightyellow”, “pink”, “purple”, “royalblue”, “salmon”, “tan”, and “turquoise” were correlated (*p*-value < 0.10) with at least three traits (Fig. 5b). Differently than what was observed in co-expression analysis of AA group, the AB-modules showed higher correlation values (negative or positive) for IMF, palmitoleic, oleic and linoleic acids.

### Hotspot 2 - rs110242967, BTA11: 10540044-12540044, *EGR4*

The “magenta” and “purple” AA-modules were correlated (*p*-value < 0.10) with at least three different traits as shown in Fig. 6a. “Purple” presented the highest correlation values (greater than 0.17) as well as a significantly correlated (*p*-value < 0.10) with eight of the 15 phenotypes studied.

“Yellow”, “brown”, “pink” and “blue” AB-modules were respectively correlated (*p*-value < 0.10) with the following traits: BFT (*r* = − 0.52), myristic (*r* = 0.50) and sum of n3 (*r* = 0.41); stearic (*r* = − 0.35), oleic (*r* = 0.45), CLA cis9 tran11 (*r* = 0.55), SFA (*r* = − 0.38); IMF (*r* = − 0.39), sum of n3 (*r* = − 0.36) and sum of n6 (*r* = − 0.42); BFT (*r* = − 0.47), CLA cis9 tran11 (*r* = 0.46) and palmitoleic (*r* = 0.37) (Fig. 6b).

### Hotspot 3 - rs134095631, BTA14: 79693309-74693309, *RUNX1T1*

“Greenyellow”, “black”, “blue”, “magenta”, “midnightblue”, “pink”, “purple”, and “salmon” AA-modules were correlated (*p*-value < 0.10) were correlated (Fig. 7a) with at least three traits. “Darkgreen”, “darkred”, “green” and “yellow” AB-modules were correlated (*p*-value < 0.1) with at least three different traits as shown in Fig. 7b. As observed for the other two hotspots, the AB-modules showed higher correlation values (negative or positive), however for different traits such as BFT, myristic, palmitoleic, oleic acids and PUFA.

### Hotspot’s functional enrichment analysis, network construction and visualization

**Fig. 8:**
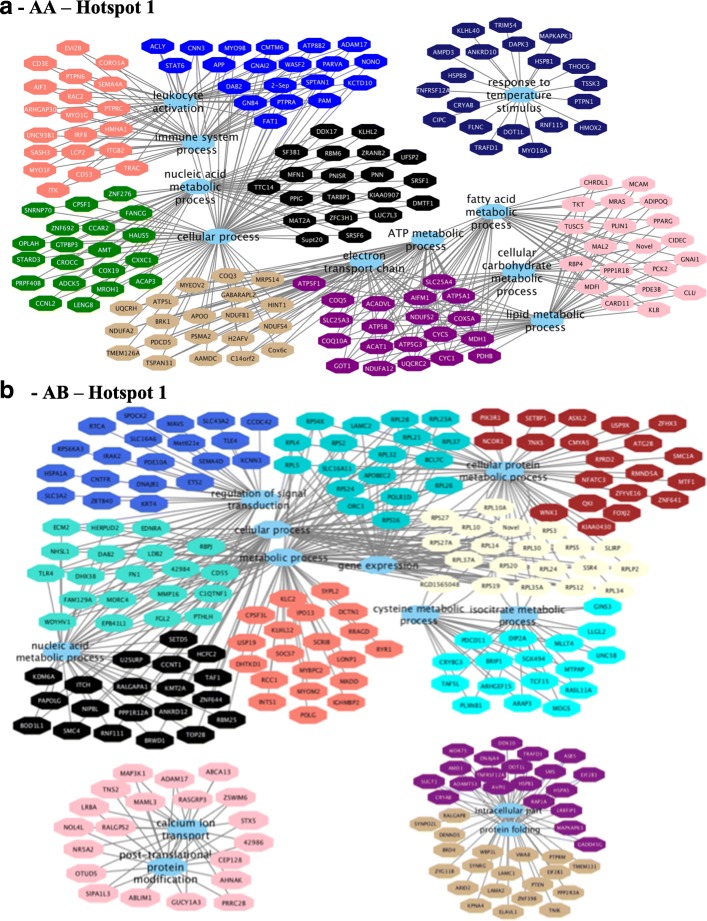
Co-expression networks show the top 20 hub genes of AA (**a**) and AB-modules (**b**) of hotspot 1 correlated (*p*-value < 0.10) with at least three different traits associated with lipid deposition and composition in skeletal muscle of Nellore steers. Colored octagons represent the hub genes within each module, and blue octagons represent the biological processes associated (FDR 5%) with the genes

**Fig. 9:**
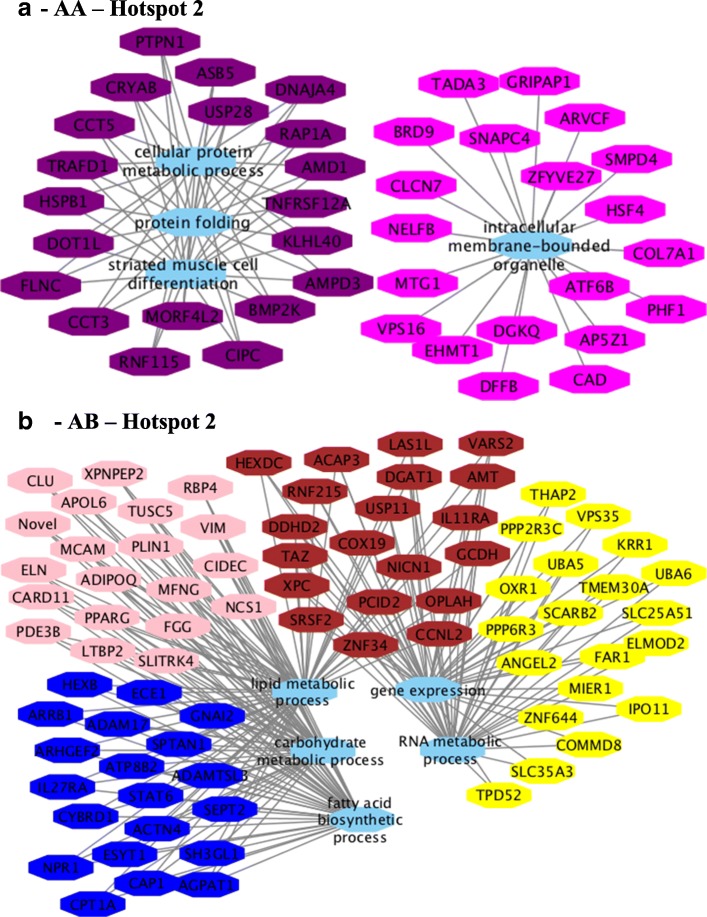
Co-expression networks show the top 20 hub genes of AA (**a**) and AB-modules (**b**) of hotspot 2 correlated (*p*-value < 0.10) with at least three different traits associated with lipid deposition and composition in skeletal muscle of Nellore steers. Colored octagons represent the hub genes within each module, and blue octagons represent the biological processes associated (FDR 5%) with the genes

**Fig. 10:**
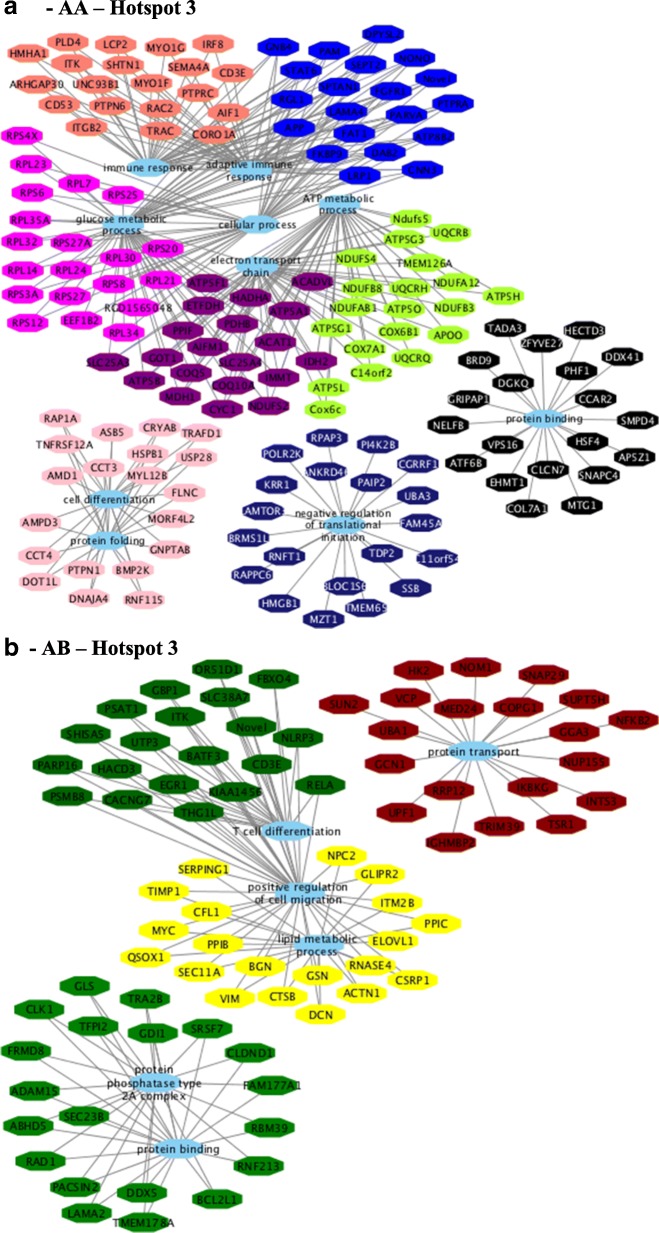
Co-expression networks show the top 20 hub genes of AA (**a**) and AB-modules (**b**) of hotspot 3 correlated (*p*-value < 0.10) with at least three different traits associated with lipid deposition and composition in skeletal muscle of Nellore steers. Colored octagons represent the hub genes within each module, and blue octagons represent the biological processes associated (FDR 5%) with the genes

Functional enrichment analysis was performed from a list of genes within each module that were significantly correlated (*p*-value < 0.10) with at least three different traits according to the Cytoscape plugin BINGO [29]. Network construction and visualization for each eQTL hotspot genotype (see Additional file 17, sheets 1, 2, 5, 6, 9, 10) (Figs. 8, 9 and 10) were performed by Cytoscape 3.5.1 [30] connecting the top 20 hub genes [most connected genes, higher values of the module membership (MM)] of each module by the common significant (FDR 5%) biological process (BP) gene ontology (GO) terms.

Functional enrichment analysis for hotspot 1 identified the following BP GO term for the AA (Fig. 8a) genotype: immune system process, lipid metabolic process, cellular carbohydrate metabolic process, fatty acid metabolic process, electron transport chain, oxidation reduction, and mitochondrial ATP synthesis coupled electron transport. For the AB (Fig. 8b) genotype, the following BP GO terms were identified: translation, gene expression, regulation of transduction, regulation of cell communication, and cellular process (see Additional file 17, sheets 3 and 4).

For hotspot 2, the following BP GO terms were identified for AA (Fig. 9a) genotype: intracellular membrane-bounded organelle and cytoplasm, protein folding, response to temperature stimulus, muscle structure development, cell differentiation, striated muscle cell differentiation and muscle cell differentiation. BP were as follows for the AB (Fig. 9b) genotype: signal transduction, lipid metabolic process, carbohydrate metabolic process, cellular lipid metabolic process, fatty acid biosynthetic process, gene expression, lipid biosynthetic process, RNA metabolic process (see Additional file 17, sheets 7 and 8).

For hotspot 3, the following BP GO terms were identified for AA (Fig. 10a) genotype: electron transport chain, ATP metabolic process, protein binding, glucose metabolic process, and immune response. BP were as follows for the AB (Fig. 10b) genotype: and positive regulation of cell migration, T cell differentiation, protein binding, and lipid metabolic process (see Additional file 17, sheets 11 and 12).

## Discussion

### Identification of eQTL and their overlap with QTL regions

To our knowledge, this is the first eQTL analysis performed for complex phenotypes such as IMF content traits in bovine species. The association analysis between each of the 461,466 SNPs and the expression level of 11,808 genes expressed in muscle identified 1268 cis-eQTLs and 10,334 trans eQTLs that affected 243 and 1453 genes, respectively. These results agree with Wittkopp and Kalay [31] that described cis regulators that affected only a few genes and trans regulators having pleiotropic effect on many genes. The higher number of trans-eQTLs than cis-eQTLs was also reported by Ramasamy et al. [32].

The overlapping test between eQTL and previous QTL regions support the hypothesis that many SNP-trait associations are mediated by changes in the expression level. Previous studies demonstrated that untranslated gene regions (UTR), such as 5′ and 3′ UTRs, introns and intergenic regions are involved in the regulation of expression [33] and that variation within these regions produce phenotypic variation [34–36].

In this study, although we used a panel of SNPs, in which most of the mutations are in introns and intergenic regions, we observed a enrichment of the intergenic regions for the identified trans-eQTLs. These findings corroborate the expected biologic function of the trans-eQTLs as potential distant regulators of gene expression [37].

Functional annotation of genes located near (2 Mb) cis and trans-eQTLs revealed biological processes GO terms such as transcription factor binding, protein binding, translation regulatory and transporter activities. Because of the presence of regulatory genes near eQTL regions, we suggest that these regions could be involved in modulation of gene expression and thus influencing quantitative traits [38, 39].

### Hotspot eQTLs and transcription factors

Cis-eQTLs are in general considered more important than trans-eQTL because of their local activity [40, 41]; however, recent studies have demonstrated that both cis- and trans-eQTLs are important to better understand the expression variation in different species [42, 43]. Among the trans-eQTL, some were associated with the expression levels of many different genes [see Additional file 3], which were defined as eQTL hotspots as described previously [44, 45]. This pleiotropic effect can be explained by the presence of a TF in the hotspot eQTL region [46]. Even though the annotation of the bovine genome for TF is not complete, we identified three annotated (JASPAR database) [47] TFs near (2 Mb) the following eQTL hotspots: USF1 (BTA3: 8117390–10117390), *EGR4* (hotspot on BTA11: 10540044–12540044) and *RUNX1T1* (BTA14: 79693309–74693309). The involvement of these TF was supported by the presence of TFBS in the promoter region of differentially expressed genes. In another study, authors also demonstrated that many of the harbored TFs identified within trans-eQTLs regions mediate the effect of inheritance of these loci on gene expression levels [46, 48]. In addition, the association test performed between these eQTL hotspots and the phenotypes confirmed the significant (FDR 5%) effect of these eQTLs on phenotypic variances of IMF content traits (Table 1).

The three annotated TFs identified near the eQTL hotspots were previously reported to be associated with lipid metabolism. *USF1* (Upstream Transcription Factor 1) encodes a member of the basic helix-loop-helix leucine zipper family and has been linked to familial combined hyperlipidemia (*FCHL*) [49]. A recent report pointed *USF1* as a new molecular link between lipid metabolism and energy expenditure, which is a potential therapeutic target for cardiometabolic disease in humans [50]. *EGR4* (Early Growth Response Protein 4) is one of the prototypes of a family of zinc-finger transcription factors, which activates the transcription of target genes whose products are required for processes such as mitogenesis and differentiation [49]. *EGR4* is an important TF in neuronal maturation and its expression is induced by cerebral ischemia and inflammation [51, 52]. Interaction of *EGR4* and fatty acids with *EGR1* and the PPAR pathway was associated with cardiovascular risk [53]. Finally, the TF *RUNX1T1* (Runt Related Transcription Factor 1 Translocation Partner 1) is a transcriptional co-repressor that acts as a negative regulator of adipogenesis [49]. A recent study reported that *RUNX1T1* is an inhibitor of brown adipogenesis (associated with a lean and healthy phenotype), which was associated with obesity and suggested that the miRNAs that downregulates this TF could be part of novel therapeutics to increase BAT (brown adipose tissue) in humans [54]. *RUNX1T1* has been also implicated in epigenetic regulation as demonstrated in genome-wide methylation study following prenatal and postnatal dietary omega-3 fatty acid supplementation in pigs, which was differentially methylated between the treatments [55].

Other important candidate regulators were also identified in the hotspot eQTLs regions, such as nuclear receptors (*NR1I3*, *NCOR*, *NRBF2*), miRNAs (bta-mir-2294, bta-mir-1584, bta-mir-2322, and bta-mir-584-3) and small nuclear RNAs (*SNORA70*e, *SNORA19* and *SNORA9*).

The nuclear receptor subfamily 1 member 3 (*NR1I3*) is an important regulator of xenobiotic, bile acid, and cholesterol/HDL metabolism, energy homeostasis [56]. While, nuclear receptor binding factor 2 (*NRBF2*) is associated with the mTORC1 activation by lysosomal cholesterol, which is directly dependent of fatty acid content [57].

The transcriptional corepressor *NCOR*, interacts with nuclear receptors and mediates the silencing of retinoid and thyroid receptors [58]. LXRs demonstrate higher affinity for *NCOR* in biochemical assays, which when up-regulated increases the synthesis of long chain FAs (PUFA). These FAs, such as palmitoleic acid, eicospentaenoic acid (EPA), and docosahexaenoic acid (DHA), are involved in anti-inflammatory activity [59].

Both small nuclear RNAs and miRNAs are non-coding regulatory RNAs, which utilize a similar set of processing enzymes. They are involved in several biological processes such as cell differentiation, cell proliferation, cell death, metabolic control, and transposon silencing [60]. It was interesting to observe that eQTL analysis also identified trans-eQTLs that act as post-transcriptional regulators.

### Correlation between the eQTL hotspots and IMF content traits

It is known that DNA polymorphisms can alter complex phenotypic traits by modulating gene expression [4, 5]. Here we showed that genetic variation can influence gene expression, but the question of how alteration of gene expression could influence the phenotype remains unanswered. To further explore this question, we performed co-expression analysis that used the expression profile data between the two most frequently observed genotypes (AA and AB) of the three eQTL hotspot regions. Furthermore, we correlated the constructed modules with the phenotypes. These regions harbored the annotated TFs and are not in LD. WGCNA has been successfully used to investigate how gene expression changes are coordinated across transcripts and how these changes are associated with phenotype [61, 62].

Our results revealed significant differences in the pattern of the network construction (modules) for AA versus AB genotypes, as well as for correlation between network module eigengene (ME) values and phenotypic traits for all the three hotspots. The change in the number of modules present for each genotype may be attributed to the significant difference in co-activation of genes expressed in skeletal muscle, potentially due to regulation or mutation in these transcription factors. This would explain the differences in module content and GO enrichment results across genotypes. These findings also support the hypothesis that re-wiring of gene expression modulates pathways that can influence the traits under study.

Recent studies showed that hub genes (highly connected) tend to play important roles in co-expressed networks (modules) acting as potential regulators [63, 64]. Many hub genes and candidate genes for phenotypic variation were identified in this study such as *TMEM39B*, *BCAR1*, *MED19*, *LIN37*, *TRIM54* and *PPAR* (see Additional file 17), were previously identified as differentially expressed genes between groups of animals with extreme values of IMF deposition and composition [17, 18]. The module-trait correlation analysis revealed connected modules and hub genes associated with BP such as immune response, leukocyte proliferation, lipid metabolism, and fatty acid biosynthesis for the different genotypes and hotspots. Some of these hub genes could be indicated as candidate biomarkers because they probably share similar functions. Second, several hub genes (*RPL4*, *RPL5*, *BCL7C*, *KCNN3*, *DNAJB1*, *COX19*, *UBA6*, *UBA5*) were associated with BP such as gene expression, nucleic acid metabolic, and RNA metabolic processes suggesting they play an important role in gene expression regulation. Based on the genotype specific differences in number of modules, module-trait relationships pattern, co-expression networks constructed, and the functional enrichment between the two genotypes, some hub genes identified in this study are plausible biomarkers for fatty acid variability such as *TMEM39B* and *PPAR*.

Genetic variation in the protein-coding region of a TF could modify the interaction of TF with TFBS. However, in our data we did not find any SNP in the coding regions of *USF1*, *EGR4* and *RUNX1T1* associated with the AA and AB genotypes, but one could not exclude the possibility that the observed effects result from linkage disequilibrium with a SNP not represented in the Illumina bovine chip. Alternatively, genetic variation in the promoter region of a TF could influence the abundance of the TF and thus alter expression of downstream genes. The low expression level of these TFs in our study could explain the lack of difference in TF gene expression between the AA and AB genotypes. Thus, further research is necessary to determine the causative mutation associated with the TF identified.

## Conclusion

We identified several regions across the genome that affect gene expression level (expression quantitative trait loci, eQTL) and overlap with QTL regions associated with the deposition and composition of IMF. Some of these regions harbor TF and control the expression of several genes (hotspots). Results obtained supported the hypothesis that eQTL analysis can be used to identify putative regulatory regions and transcription factors associated with important phenotypic traits that are controlled by modulation of gene expression profile.

## Methods

### Animals, phenotype and genotype data

The animals (*n* = 192), phenotype and genotype data used in this study was comprised of Nellore steers sired by 34 unrelated sires, selected to represent the main genealogies used in Brazil according to the National Summary of Nellore produced by the Brazilian Association of Zebu Breeders (ABCZ) and National Research Center for Beef Cattle to ensure compliance with international guidelines for animal welfare as described previously by Cesar et al. [15]. A captive bolt pistol was used for stunning the animals prior to slaughter. SNPs with call rate ≤ 95%, minor allele frequency (MAF) ≤ 5%, those located on sex chromosomes or not mapped in the *Bos taurus* UMD 3.1 assembly were removed. The MAF threshold was chosen based on the sample size in order to minimize the number of false-positive and false-negative associations [65]. After filtering, a total of 461,643 SNP was utilized in eQTL mapping.

### RNA extraction and sequencing

Total RNA was extracted from 100 mg of frozen LD muscle from 192 animals that were collected at slaughter using the TRIzol reagent (Life Technologies, Carlsbad, CA). RNA integrity was verified by Bioanalyzer 2100 (Agilent, Santa Clara, CA, USA). Only samples with RIN > 8 were used. A total of 2 μg of total RNA from each sample was used for library preparation according to the protocol described in the TruSeq RNA Sample Preparation kit v2 guide (Illumina, San Diego, CA). Average library sizes were estimated using the Agilent Bioanalyzer 2100 (Agilent, Santa Clara, CA, USA) and quantified using quantitative PCR with the KAPA Library Quantification kit (KAPA Biosystems, Foster City, CA, USA). Quantified samples were diluted and pooled (three pools of six samples each). Three lanes of a sequencing flowcell, using the TruSeq PE Cluster kit v3-cBot-HS kit (Illumina, San Diego, CA, USA), were clustered and sequenced using HiScanSQ equipment (Illumina, San Diego, CA, USA) with a TruSeq SBS Kit v3-HS (200 cycles), according to manufacturer’s instructions. Sequencing analyses were performed at the Genomics Center at ESALQ, Piracicaba, São Paulo, Brazil.

Sequencing adaptors and low-complexity reads were removed in an initial data-filtering step. Quality control and read statistics were estimated with FASTQC version 0.10.1 software [https://www.bioinformatics.babraham.ac.uk/projects/fastqc/]. RNA-Seq by Expectation Maximization (RSEM) approach was performed to estimate the number of fragments originating from each gene in each replicate library, which is capable of handling reads that map ambiguously between isoforms and genes, and minimize the differences in total read counts across samples (normalization procedure) [66]. The UMD3.1 *Bos taurus* assembly available at Ensembl [http://www.ensembl.org/Bos_taurus/Info/Index/] was used as reference genome.

### Identification of eQTL and hotspot regions

The Matrix eQTL R package [67] was used to identify associations between genetic variation from genotype (SNPchip) and gene expression (RNA-Seq) [68]. Contemporary group (including farm, year and slaughter date) and lane were included in the model as fixed effects and age as a covariate. Markers associated with variation in gene expression that were within 1 Mb of the gene were defined as cis-eQTLs (local variants), while markers more than 1 Mb from the gene were defined as trans-eQTLs (distant variants). Matrix eQTL tests the association between each marker (SNP) and each gene assuming the effect of genotype as additive, performs a separate test for each pair (marker and gene) and corrects for multiple tests by calculating false discovery rate (FDR) [69]. The estimated effect size (slope coefficient) and the genetic variance explained by the markers was also provided according the Matrix eQTL package [66]. eQTL hotspots (markers that affect the gene expression level for many genes) were identified by permuting the distribution of eQTLs across the genome after 1000 permutations. A hotspot threshold was identified that corresponded to the 95th percentile of the value. Linkage disequilibrium (LD) analysis and visualization by PLINK v.1.07 [70] and Haploview [71], respectively, were used to check if the hotspots were in LD with each other and if so to select just one as the eQTL hotspot. The hotspot region was defined as a 4 Mb window around the hotspot eQTL, i.e. 2 Mb extended to each side of the hotspot eQTL.

### Association test between eQTL hotspots and the phenotypes

The analysis of variance (ANOVA) model containning contemporary group (including farm, year and slaughter date) and lane as fixed effects and age as a covariate and was applied to test for association between a given SNP and a corresponding phenotype. Evidence of population stratification was not identified in this population based on previous results reported by our group [72]. Therefore, it was not included in the model to detect eQTL. The statistical test was performed by R software and applied to verify the effect of the eQTL hotspots identified in this study on the phenotypes of interest. The correction for multiple tests was applied by calculating false discovery rate (FDR 5%).

### Overlap statistics (eQTLs / QTLdb)

Overlap analysis was carried out using the Bioconductor package regioneR [24]. The package implements a general framework for testing overlaps of genomic regions based on permutation sampling. We repeatedly sampled random regions (*N* = 1000 permutations) from the UMD_3.1 genome assembly matching size and chromosomal distribution of the detected eQTLs. This test was performed for QTL class, QTL associated with traits of production and quality of carcass and meat, and QTL previously reported by our group [15] associated with the traits of interest in this study. In every permutation, the overlap with the cattle QTLdb [23] was recomputed based on the total genomic size in Mb that was overlapped.

### Annotation and functional annotation of the eQTLs

The eQTL annotations were performed using Ensembl Variant Effect Predictor, a free toolset for the analysis, annotation, and prioritization of genomic variants in coding and non-coding regions [73]. The reference genome assembly used was UMD3.1 *Bos Taurus* from Ensembl data bank [73]. With this set of tools, the location of an eQTL in relation to a gene can be defined as outside of the gene, in the coding sequence, or in untranslated regions (UTR). The functional impact was determined for those eQTLs that were localized in the coding sequences. Functional enrichment analyses were performed with Protein ANalysis THrough Evolutionary Relationships (PANTHER) [26] using the list of the genes harbored in 4 Mb eQTL regions (hotspot, cis and trans). The statistical over-representation test by PANTHER was used to obtain the gene ontology association (biological processes and protein classes) from a given list of genes. That test was performed to compare a list of reference genes (background, all genes expressed in skeletal muscle identified in this study) to a list of genes harbored within 4 Mb eQTL regions, and determine if a particular class of gene ontology (GO) biological processes were over-represented or under-represented (nominal *p*-value ≤0.05).

### Transcription factor binding site searching

Annotated transcription factors (TFs) by JASPAR CORE database [42] were searched within the eQTL hotspot region, and the transcription factor binding sites (TFBSs) of these TFs were searched using LASAGNA-Search 2.0 [28]. LASAGNA-Search 2.0 is an integrated web tool based on the algorithm Length-Aware Site Alignment Guided by Nucleotide Association, which allows the identification of TFBS from a list of target genes. To perform the LASAGNA-Search 2.0 program, the TFBSs and position-specific scoring matrix (PSSM) were collected from JASPAR CORE database; the name of the TFs were chosen based on the *Bos taurus* genome annotation; and the list of target genes was the list of gene affected by the eQTL hotspots identified herein. This method used by LASAGNA 2.0 can distinguish true binding sites from other non-functional sites with similar sequences by giving a weighted match to any given substring (combinations) of fixed length. The TFBS were searched in 1500 bp of length of promoter region obtained from Biomart tool of Ensembl website [http://www.ensembl.org/biomart] of those genes that were affected by eQTL hotspot for the specific TF.

### Association between eQTL hotspots and traits by co-expression network analysis

Hotspot eQTLs were chosen that had annotated TFs within them to associate the hotspot eQTLs with the traits of interest (IMF deposition and composition). WGCNA (Weighted Gene Correlation Network Analysis), which is a systems biology network method that describes the correlation patterns among all expressed genes across samples, was performed by WGCNA R package [74]. This approach was used to identify the differences in co-expression networks between hotspot eQTL genotypes (AA and AB, MAF > 0.05). Gene networks were constructed separately for each of the two most frequent genotypes, which were assigned an arbitrary color. For WGCNA analysis, the correlation matrix was built using the absolute value of the Pearson correlation coefficient between all gene pairs across all samples. The Pearson correlation matrix was subsequently transformed into an adjacency matrix (A) using a power function based on scale-free topology criterion, as described [75]. A soft threshold power of 6 with scale free fitting index of 0.9 was applied to calculate the adjacency matrix. TOM-based dissimilarity (1-TOM) was used for module identification using Dynamic Tree Cut algorithm with cutreeDynamic function in WGCNA package [74] and defining the deep split = 2 and minimum module size = 30. To make the genotype networks comparable, for each eQTL hotspot, we scaled the TOM (Topology Overlap Matrix) connectivity’s in genotype with the minimum number of data such that its 95th percentile equals the 95th percentile of the genotype with maximum number of data, as described by Langfelder and Horvath [76]. To quantify co-expression similarity of entire modules, we calculated their eigengene values using moduleEigengenes function in WGCNA package and clustered them based on their correlation using height cut of 0.25, which corresponded to a correlation of 0.75, to merge similar modules [76]. All other WGCNA parameters remained at their default settings. Grey-colored modules contained all genes that were not part of any module. The associations between individual genes with traits of interest (fat deposition and composition of intramuscular fat, adjusted phenotype as described by Cesar et al. [18] was quantified by the Gene Significance (GS) approach, which was defined as the correlation (the absolute value) between the gene and the trait of interest. The quantitative measure of module membership (MM) was defined as the correlation of the module eigengene and the gene expression profile. With these assumptions, the similarity of all genes was quantified to every module. The *p*-value threshold applied in this correlation analysis was *p*-value < 0.10 based on previous studies that used the same approach [19, 77].

**Fig. 11:**
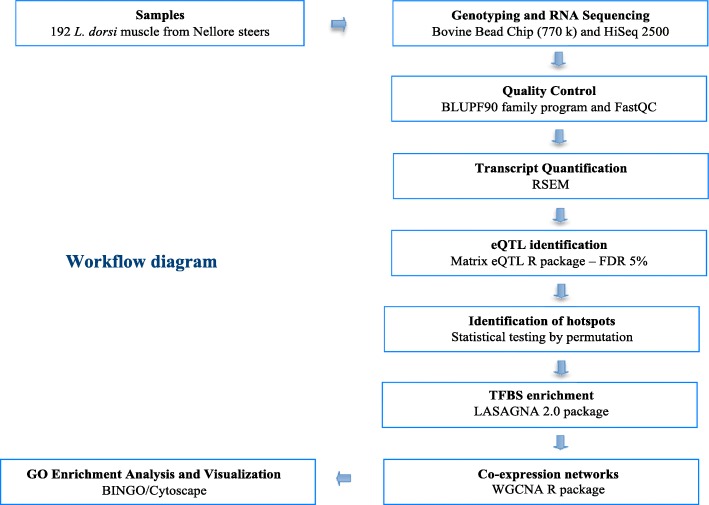
A workflow diagram of eQTL study in Nellore population

Gene Ontology (GO) annotation from a list of genes within of each module significantly correlated (*p*-value < 0.10) with at least three different traits by Cytoscape plugin BINGO [25] using the latest update of gene ontology annotation database (GOA) [78]. The statistical significance of GO term enrichment was measured by a hypergeometric test using the genes in the whole network as the background (all genes expressed in skeletal muscle). The Benjamini and Hochberg [69] correction (p-adjusted) was used to correct for multiple testing. Only GO terms that were significantly over-represented (p-adjusted ≤0.05) were reported. This functional enrichment analysis was followed by network construction using the hub genes to support the hypothesis that the expression pattern of the modules correlated to the phenotypes can influence the IMF content traits. The construction and visualization of the networks for each eQTL hotspot genotype were performed by Cytoscape 3.5.1 [30] connecting the top 20 hub genes [most connected genes, higher values of the module membership (MM)] of each module by the common significant (FDR 5%) BP. A workflow diagram of this study is shown in Fig. 11.

## Additional files

Additional file 1: Descriptive statistics for backfat thickness (BFT) and IMF deposition and composition traits in Nellore steers. (DOCX 61 kb)

Additional file 2: QQ-plot of the distribution of *p*-values for cis-eQTLs (red line) and trans-eQTL (blue line) using mRNA sequencing and animal genotype data. The top horizontal grey line denotes a 5% false discovery rate significance threshold for trans-eQTLs and the bottom one for cis-eQTLs. (DOCX 221 kb)

Additional file 3: All information about the cis and trans-eQTLs identified from RNA-Seq and genotyping data of Nellore steers population. (XLSX 2154 kb)

Additional file 4: Variant annotation by VEP tool for cis-eQTLs (A) and trans-eQTLs (B) identified across the whole Nellore genome. (DOCX 244 kb)

Additional file 5: Variant effect annotation of cis-eQTLs by VEP tool. (XLSX 212 kb)

Additional file 6: Variant effect annotation of trans-eQTLs by VEP tool. (XLSX 262 kb)

Additional file 7: A. Shown are the number of overlaps (y-axis) between the detected eQTLs (set A) and known QTLs from Cattle QTLdb (set B) in four overlap categories: AinB denotes an eQTL encompassed by / completely contained in a QTL (and vice versa for BinA). On the other hand, AleftB and ArightB denote partial overlap of an eQTL and with the left or the right part of a QTL, respectively. B. QTLs classes that overlap with eQTLs regions from Cattle QTLdb. Red bars correspond to the mean overlap size (in Mb) of the eQTL regions that was observed for eQTL regions for respective trait (y-axis), while the cyan bars indicate the mean overlap size (in Mb) estimated after 1000× random resamplings (x-axis). The error bars indicates the standard deviation, while permutation *p-*values are listed on the right. C. QTLs associated with beef production, carcass and beef quality that overlap with eQTLs regions. Red bars correspond to the mean overlap size (in Mb) of the eQTL regions that was observed for eQTL regions for respective trait (y-axis), while the cyan bars indicate the mean overlap size (in Mb) estimated after 1000× random resamplings (x-axis). The error bars indicates the standard deviation, while permutation *p-*values are listed on the right. (DOCX 171 kb)

Additional file 8: eQTL regions that overlap with QTLdb regions identified from the same dataset. (XLSX 43 kb)

Additional file 9: Haploview visualization of linkage disequilibrium (LD) around two eQTL hotspots. The hotspots identified on chromosome 11 (A) and chromosome 28 (B) are marked in red, and the D’ values estimated between the hotspots. D’ value represent the percentage of the time that the both markers are co-inherited. D’ prime values of 1.0 are not shown (the box is empty). The intensity of red color indicates the D’ values estimated between the hotspot. (DOCX 954 kb)

Additional file 10: R-squared values of linkage disequilibrium between each pair of hotspot eQTLs. (XLSX 54 kb)

Additional file 11: Association test between hotspot eQTLs and IMF content-traits. (XLSX 57 kb)

Additional file 12: Biological processes identified using the list of genes harbored within 2 Mb cis (A), trans (B) and hotspot (C) eQTL regions as the input gene list file. The values shown in the pie chart are the percentage of genes classified to each GO term over the total number of genes in the list used. (DOCX 599 kb)

Additional file 13: Top ten transcription factor binding sites (TFBSs) over-represented in promoter sequence of genes that were associated with *EGR4*. (XLSX 49 kb)

Additional file 14: Top ten transcription factor binding sites (TFBSs) over-represented in promoter sequence of genes that were associated with RUNX1T1. (XLSX 54 kb)

Additional file 15: Top ten transcription factor binding sites (TFBSs) over-represented in promoter sequence of genes that were associated with *USF1*. (XLSX 91 kb)

Additional file 16: Over-represented TFBS motifs of: (A) *EGR4* (*NGFI-C* aliases), (B) *RUNX1* and (C) *USF1* in genes into hotspot eQTL regions. (DOCX 240 kb)

Additional file 17: Co-expression network results and functional enrichment. (XLSX 21960 kb)

## Abbreviations

BFT: Backfat thickness
CLA: Conjugated linoleic acid
CLA-c9t11: Conjugated linoleic acid cis 9, trans 11
eQTL: Expression quantitative trait loci
FDR: False discovery rate
GO: Gene Ontology
LASAGNA: (Length-Aware Site Alignment Guided by Nucleotide Association)
LD: Linkage disequilibrium
MUFA: Monounsaturated fatty acid
NGS: Next generation sequencing
PUFA: Polyunsaturated fatty acid
QTL: Quantitative trait loci
RNA-Seq: RNA sequencing
SFA: Saturated fatty acid
TF: Transcription factor
UTR: Untranslated region
WGCNA: Weighted gene correlation network analysis
