# Supplementary material for: Identification of putative regulatory regions and transcription factors associated with intramuscular fat content traits
Source: BMC Genomics. 2018 Jun 27;19:499. doi: 10.1186/s12864-018-4871-y (PMC6020320; doi:10.1186/s12864-018-4871-y)
Supplement: Supplementary file 1 — Descriptive statistics for backfat thickness (BFT) and IMF deposition and composition traits in Nellore steers. (DOCX 61 kb) [file 12864_2018_4871_MOESM1_ESM.docx]

Additional file 1. Descriptive statistics for backfat thickness (BFT) and IMF deposition and composition traits in Nellore steers.

| **Traits** | **Average** | **Minimum** | **Maximum** | **Median** | **STDDV** |
| --- | --- | --- | --- | --- | --- |
| **BFT (mm)** | 6.86 | 2 | 15 | 7 | 2.32 |
| **IMF (%)** | 2.93 | 0.65 | 5.59 | 2.82 | 0.97 |
| **Myristic (%)** | 3.47 | 0.003 | 5.25 | 3.44 | 0.61 |
| **Myristoleic (%)** | 0.97 | 0.41 | 2.06 | 0.93 | 0.28 |
| **Palmitic (%)** | 26.61 | 0.26 | 42.53 | 26.27 | 3.71 |
| **Palmitoleic (%)** | 3.29 | 0.61 | 4.94 | 3.39 | 0.76 |
| **Stearic (%)** | 15.07 | 9.79 | 32.77 | 14.52 | 3.03 |
| **Oleic (%)** | 37.74 | 19.94 | 50.98 | 37.6 | 5.06 |
| **Linoleic (%)** | 0.06 | 0.001 | 0.14 | 0.06 | 0.03 |
| **CLAc9t11 (%)** | 0.22 | 0.08 | 0.42 | 0.22 | 0.07 |
| **SFA (%)** | 47.26 | 35.27 | 72.37 | 46.72 | 5.38 |
| **MUFA (%)** | 48.19 | 25.31 | 60.11 | 48.75 | 5.32 |
| **PUFA (%)** | 2.91 | 0.95 | 6.01 | 2.85 | 0.88 |
| **Sum_n3 (%)** | 0.18 | 0.03 | 0.52 | 0.13 | 0.12 |
| **Sum-n6 (%)** | 0.17 | 0 | 0.44 | 0.16 | 0.07 |
