# Supplementary material for: Identification of putative regulatory regions and transcription factors associated with intramuscular fat content traits
Source: BMC Genomics. 2018 Jun 27;19:499. doi: 10.1186/s12864-018-4871-y (PMC6020320; doi:10.1186/s12864-018-4871-y)
Supplement: Supplementary file 2 — QQ-plot of the distribution of p-values for cis-eQTLs (red line) and trans-eQTL (blue line) using mRNA sequencing and animal genotype data. The top horizontal grey line denotes a 5% false discovery rate significance threshold for trans-eQTLs and the bottom one for cis-eQTLs. (DOCX 221 kb) [file 12864_2018_4871_MOESM2_ESM.docx]

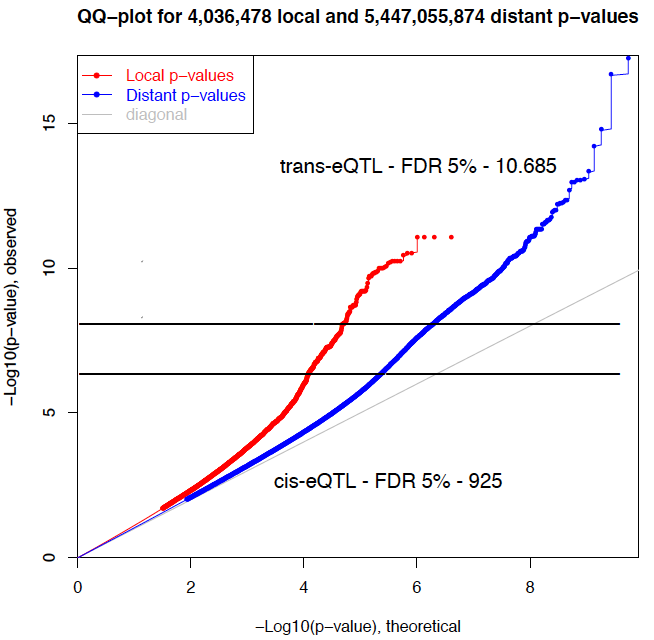


trans-eQTL (FDR 5%): 10,334

cis-eQTL (FDR 5%): 1,268

Additional file 2. QQ-plot of the distribution of p-values for cis-eQTLs (red line) and trans-eQTL (blue line) using mRNA sequencing and animal genotype data. The top horizontal grey line denotes a 5% false discovery rate significance threshold for trans-eQTLs and the bottom one for cis-eQTLs.
