# Supplementary material for: Identification of putative regulatory regions and transcription factors associated with intramuscular fat content traits
Source: BMC Genomics. 2018 Jun 27;19:499. doi: 10.1186/s12864-018-4871-y (PMC6020320; doi:10.1186/s12864-018-4871-y)
Supplement: Supplementary file 4 — Variant annotation by VEP tool for cis-eQTLs (A) and trans-eQTLs (B) identified across the whole Nellore genome. (DOCX 244 kb) [file 12864_2018_4871_MOESM4_ESM.docx]

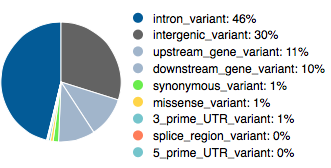

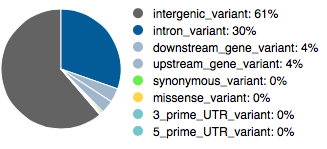


A

B

Additional file 4. Variant annotation by VEP tool for cis-eQTLs (A) and trans-eQTLs (B) identified across the whole Nellore genome.
