# Supplementary material for: Identification of putative regulatory regions and transcription factors associated with intramuscular fat content traits
Source: BMC Genomics. 2018 Jun 27;19:499. doi: 10.1186/s12864-018-4871-y (PMC6020320; doi:10.1186/s12864-018-4871-y)
Supplement: Supplementary file 7 — A. Shown are the number of overlaps (y-axis) between the detected eQTLs (set A) and known QTLs from Cattle QTLdb (set B) in four overlap categories: AinB denotes an eQTL encompassed by / completely contained in a QTL (and vice versa for BinA). On the other hand, AleftB and ArightB denote partial overlap of an eQTL and with the left or the right part of a QTL, respectively. B. QTLs classes that overlap with eQTLs regions from Cattle QTLdb. Red bars correspond to the mean overlap size (in Mb) of the eQTL regions that was observed for eQTL regions for respective trait (y-axis), while the cyan bars indicate the mean overlap size (in Mb) estimated after 1000× random resamplings (x-axis). The error bars indicates the standard deviation, while permutation p-values are listed on the right. C. QTLs associated with beef production, carcass and beef quality that overlap with eQTLs regions. Red bars correspond to the mean overlap size (in Mb) of the eQTL regions that was observed for eQTL regions for respective trait (y-axis), while the cyan bars indicate the mean overlap size (in Mb) estimated after 1000× random resamplings (x-axis). The error bars indicates the standard deviation, while permutation p-values are listed on the right. (DOCX 171 kb) [file 12864_2018_4871_MOESM7_ESM.docx]

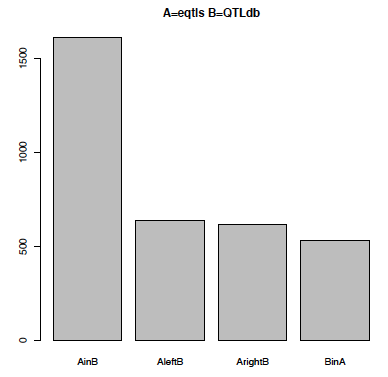


Additional file 7A.  Shown are the number of overlaps (y-axis) between the detected eQTLs (set A) and known QTLs from Cattle QTLdb (set B) in four overlap categories:

AinB denotes an eQTL encompassed by / completely contained in a QTL (and vice versa for BinA). On the other hand, AleftB and ArightB denote partial overlap of an eQTL and with the left or the right part of a QTL, respectively.


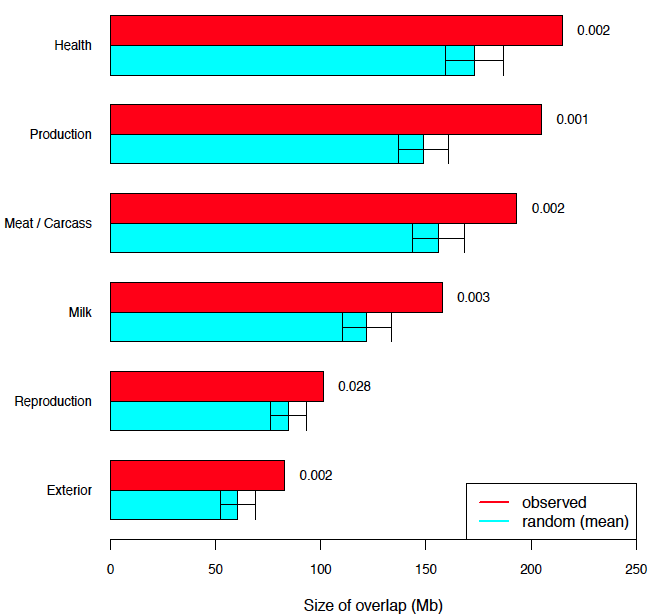


#

Additional file 7B. QTLs classes that overlap with eQTLs regions from Cattle QTLdb. Red bars correspond to the mean overlap size (in Mb) of the eQTL regions that was observed for eQTL regions for respective trait (y-axis), while the cyan bars indicate the mean overlap size (in Mb) estimated after 1000x random resamplings (x-axis). The error bars indicates the standard deviation, while permutation *p-*values are listed on the right.


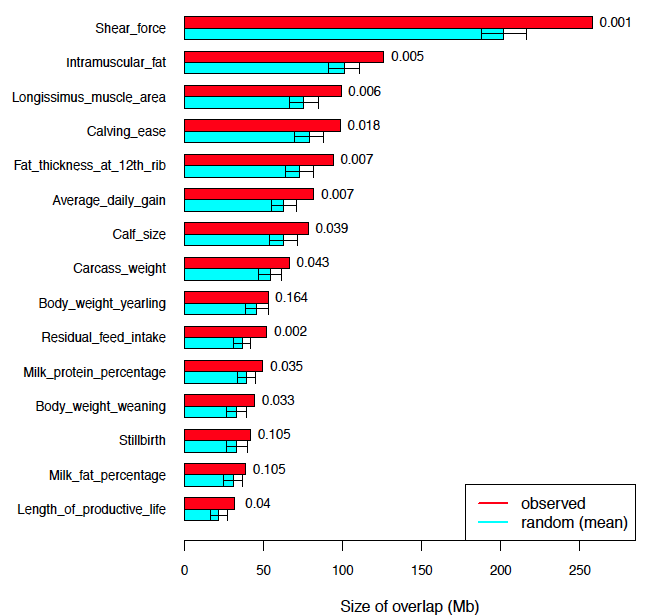


Additional File 7C. QTLs associated with beef production, carcass and beef quality that overlap with eQTLs regions. Red bars correspond to the mean overlap size (in Mb) of the eQTL regions that was observed for eQTL regions for respective trait (y-axis), while the cyan bars indicate the mean overlap size (in Mb) estimated after 1000x random resamplings (x-axis). The error bars indicates the standard deviation, while permutation *p-*values are listed on the right.
