# Supplementary material for: Identification of putative regulatory regions and transcription factors associated with intramuscular fat content traits
Source: BMC Genomics. 2018 Jun 27;19:499. doi: 10.1186/s12864-018-4871-y (PMC6020320; doi:10.1186/s12864-018-4871-y)
Supplement: Supplementary file 9 — Haploview visualization of linkage disequilibrium (LD) around two eQTL hotspots. The hotspots identified on chromosome 11 (A) and chromosome 28 (B) are marked in red, and the D’ values estimated between the hotspots. D’ value represent the percentage of the time that the both markers are co-inherited. D’ prime values of 1.0 are not shown (the box is empty). The intensity of red color indicates the D’ values estimated between the hotspot. (DOCX 954 kb) [file 12864_2018_4871_MOESM9_ESM.docx]

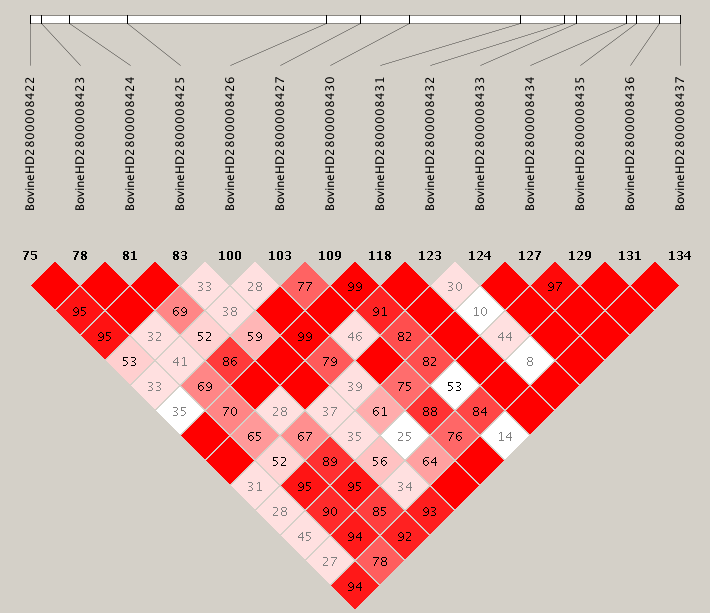


B


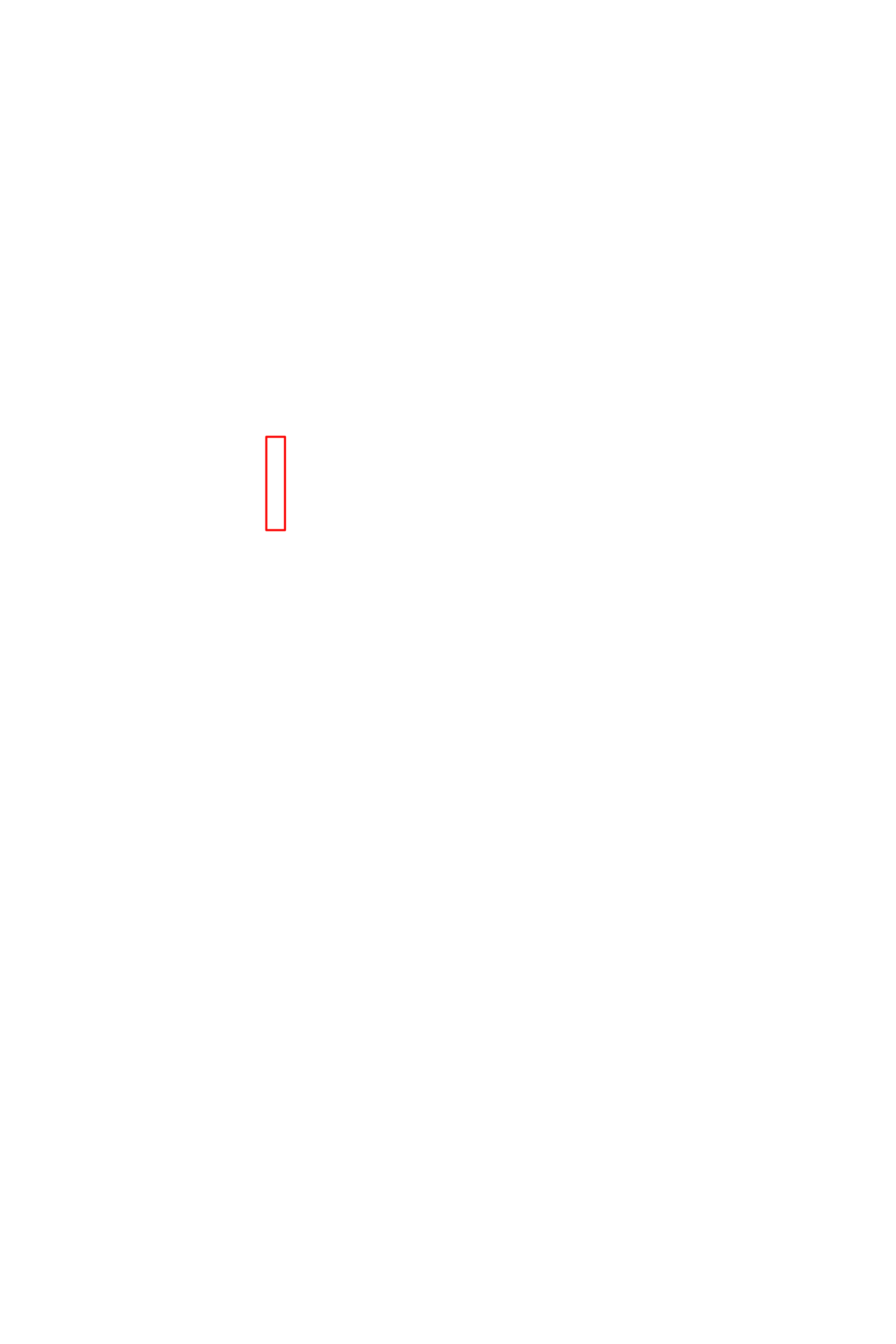


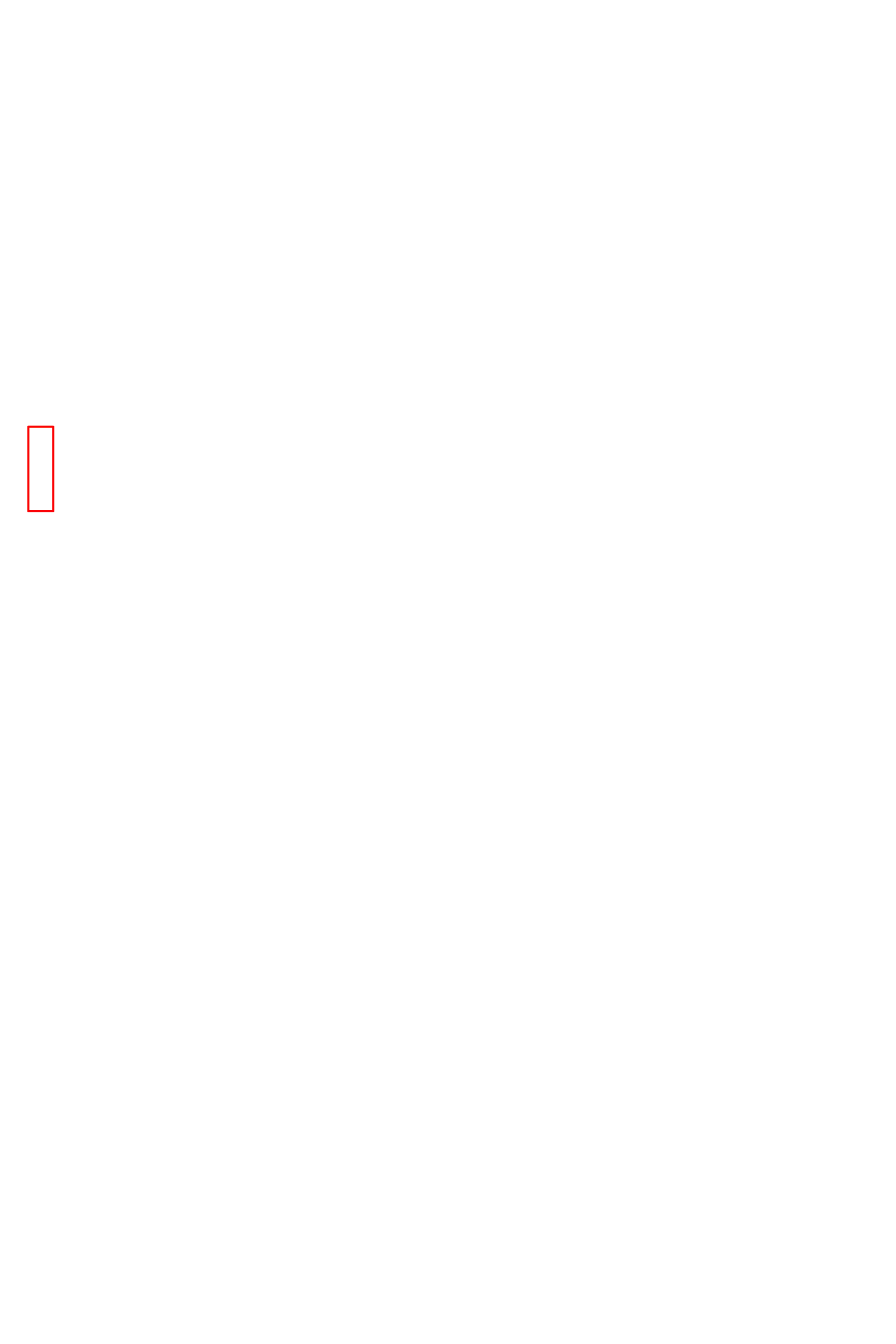


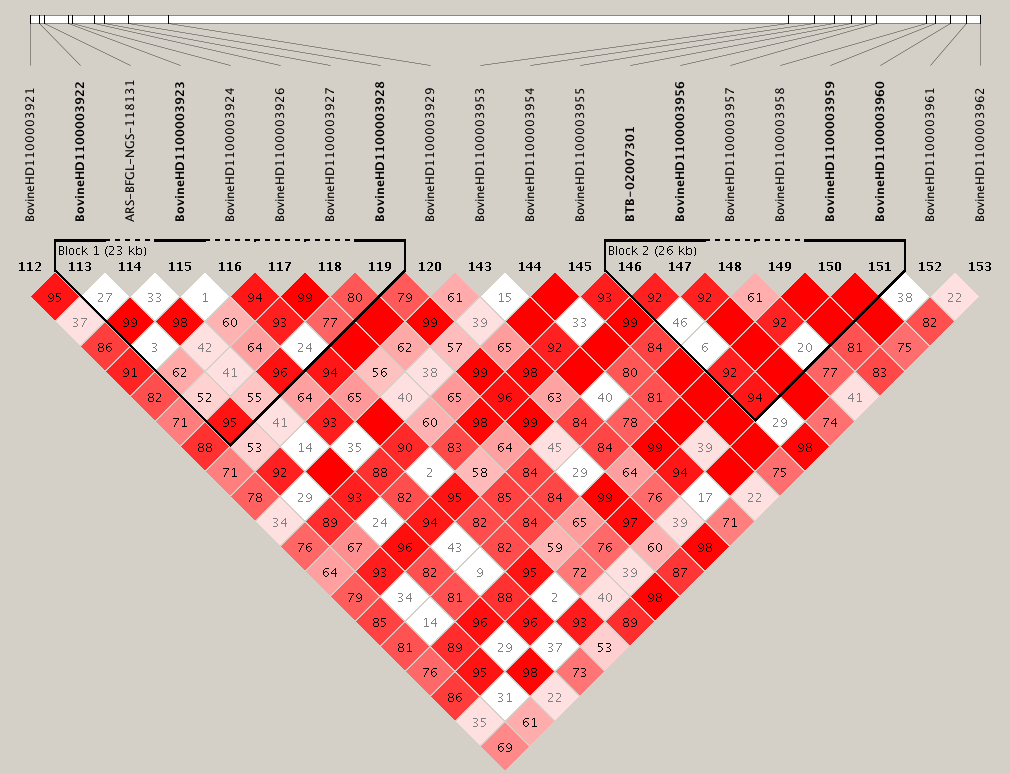


A


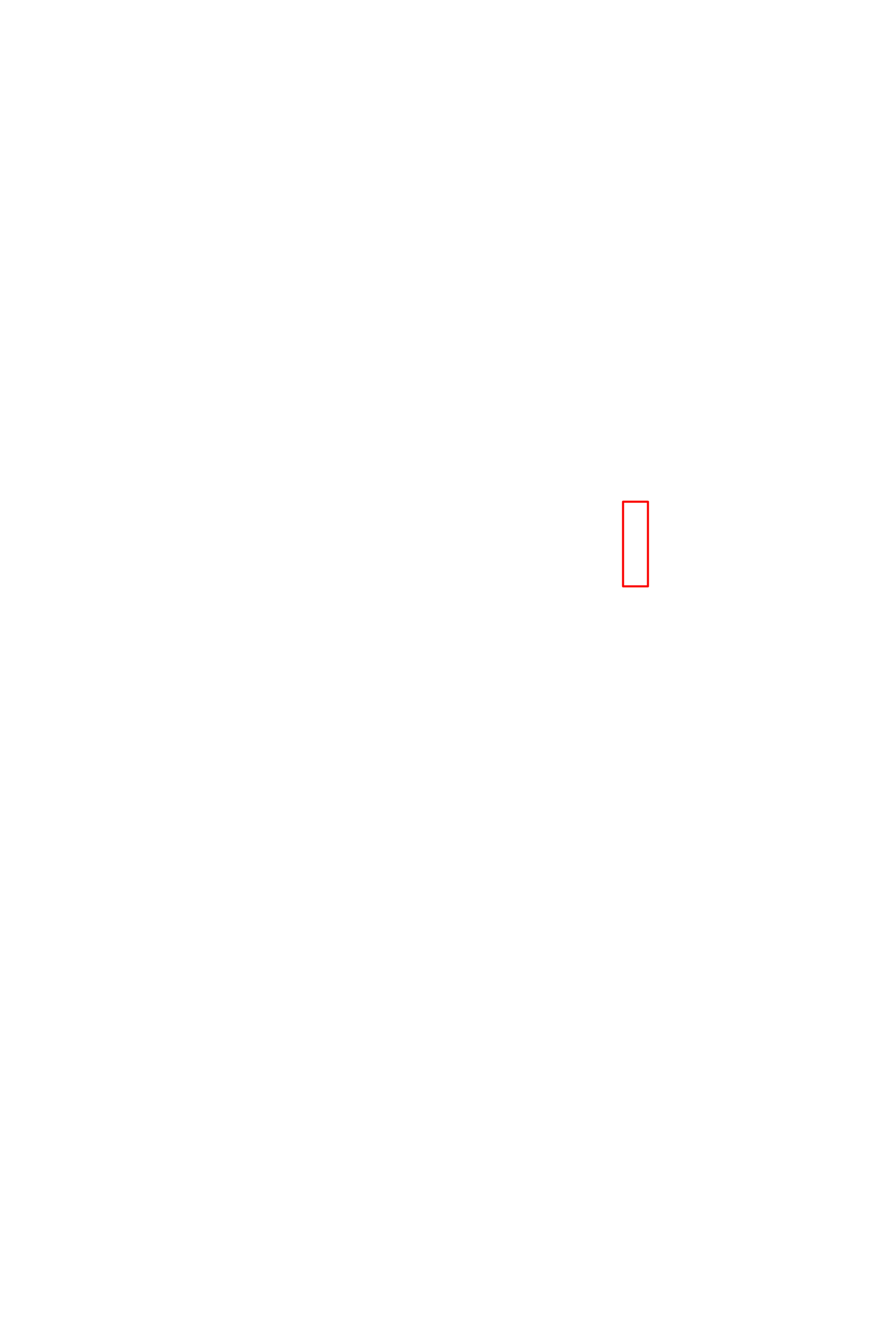


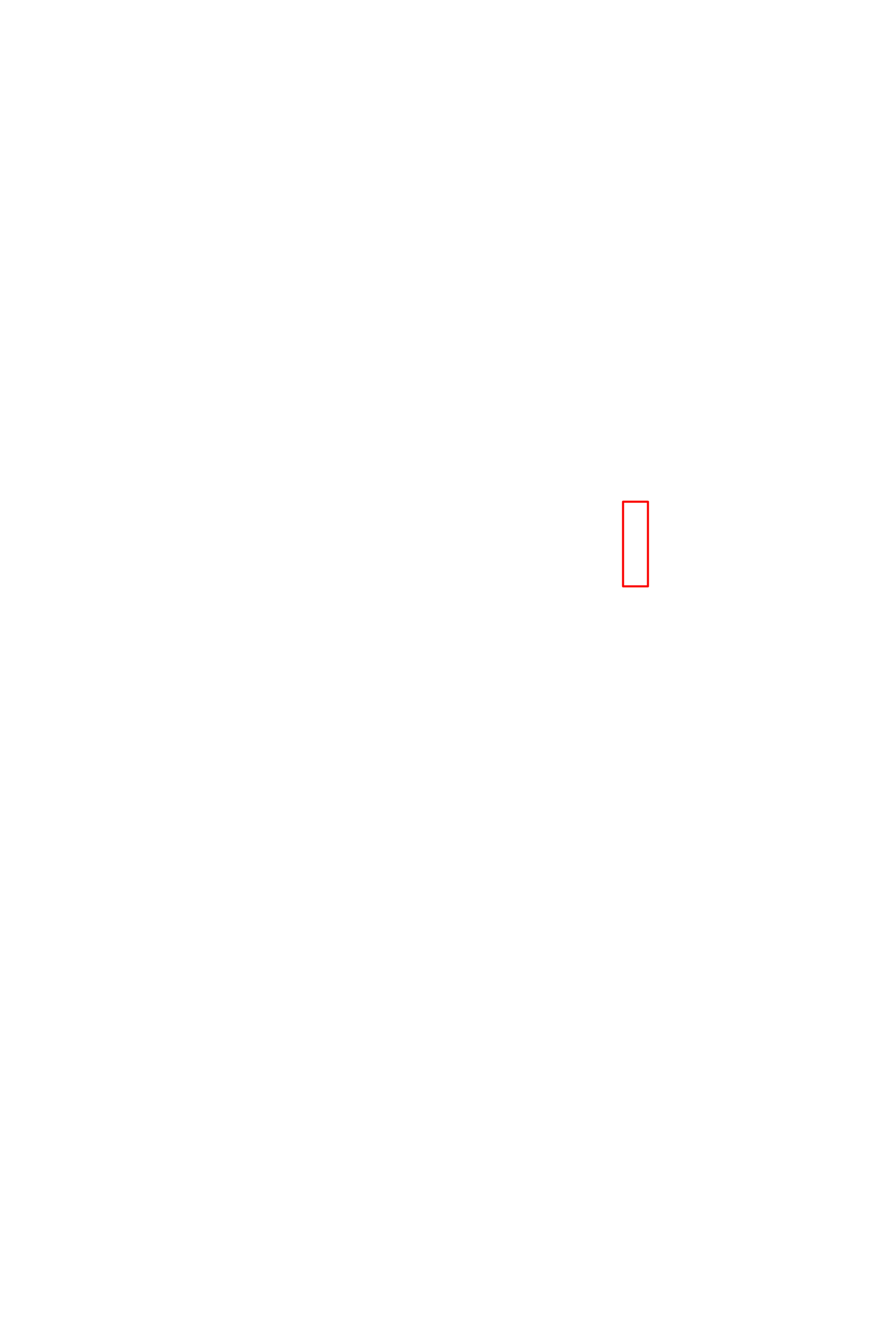


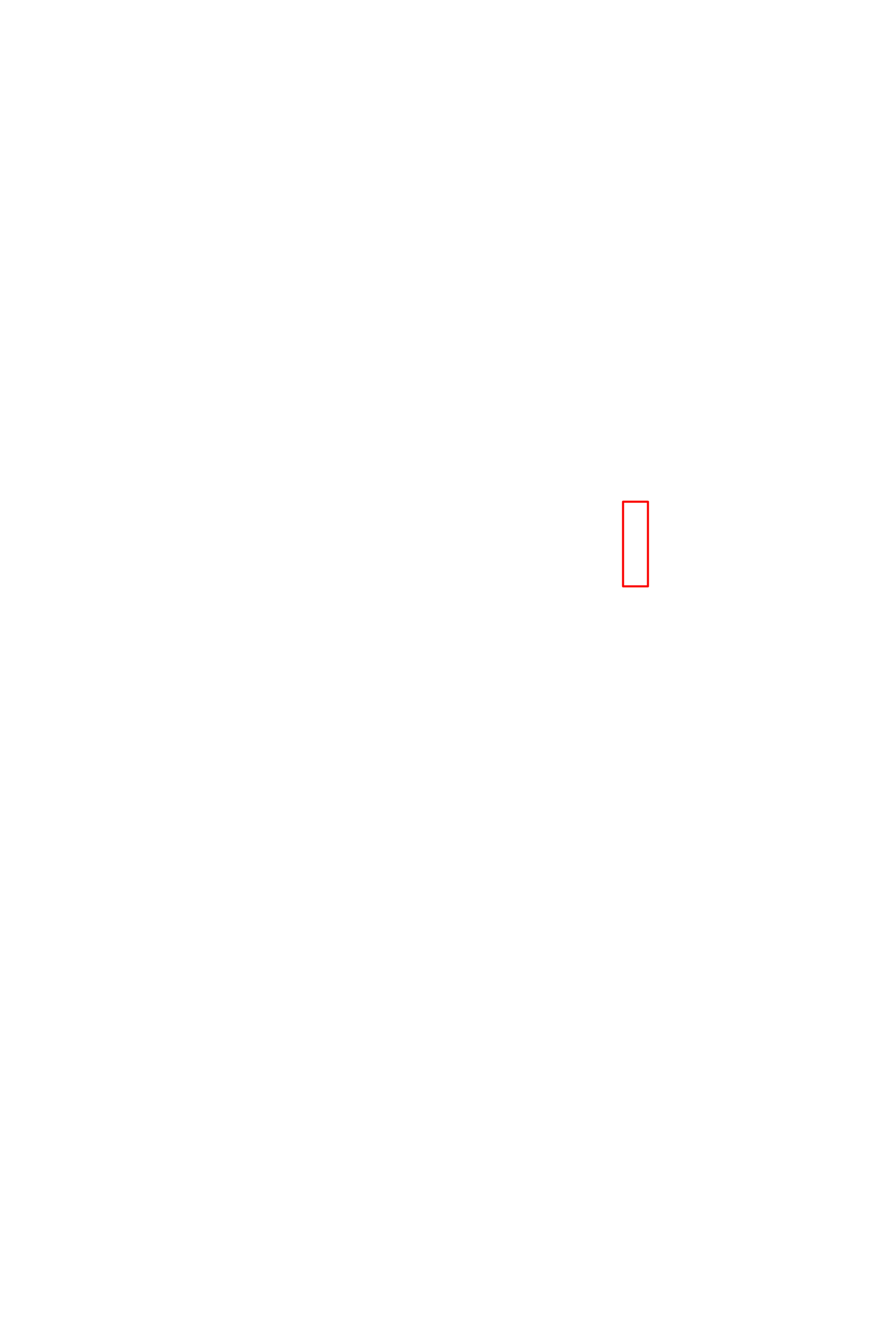


Additional file 9. Haploview visualization of linkage disequilibrium (LD) around two eQTL hotspots. The hotspots identified on chromosome 11 (A) and chromosome 28 (B) are marked in red, and the D’ values estimated between the hotspots. D’ value represent the percentage of the time that the both markers are co-inherited. D’ prime values of 1.0 are not shown (the box is empty). The intensity of red color indicates the D’ values estimated between the hotspot.
