# Supplementary material for: Identification of putative regulatory regions and transcription factors associated with intramuscular fat content traits
Source: BMC Genomics. 2018 Jun 27;19:499. doi: 10.1186/s12864-018-4871-y (PMC6020320; doi:10.1186/s12864-018-4871-y)
Supplement: Supplementary file 12 — Biological processes identified using the list of genes harbored within 2 Mb cis (A), trans (B) and hotspot (C) eQTL regions as the input gene list file. The values shown in the pie chart are the percentage of genes classified to each GO term over the total number of genes in the list used. (DOCX 599 kb) [file 12864_2018_4871_MOESM12_ESM.docx]

**B**

**A**


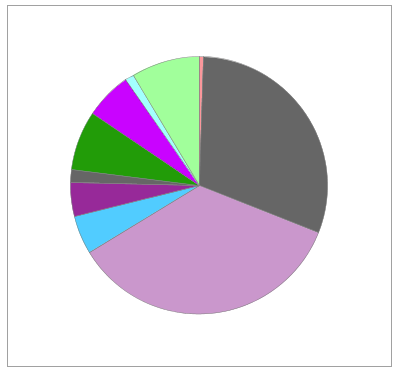

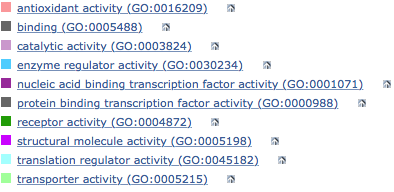


**30,5%**

**7,5%**

**5,9%**

**8,6%**

**4,3%**

**4,8%**

**35,3%**

**1,6%**

**1,1%**

**0,5%**


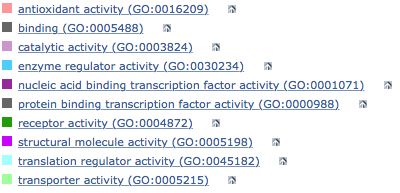

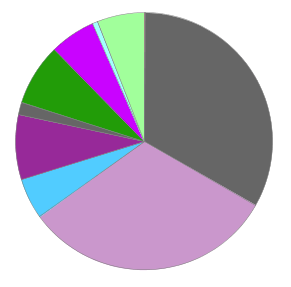


**31,8%**

**5,8%**

**7,7%**

**8,2%**

**33,1%**

**5,2%**

**5,8%**

**1,6%**

**0,7%**

**C**

**
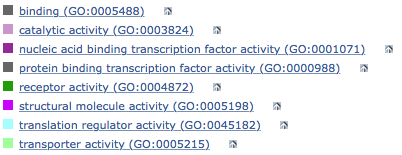

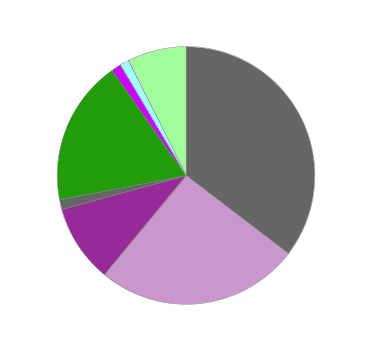
**

**35,4%**

**1,2%**

**7,3%**

**18,3%**

**9.8%**

**25,6%**

**1,2%**

**1,2%**

Additional file 12. Biological processes identified using the list of genes harbored within 2 Mb cis (A), trans (B) and hotspot (C) eQTL regions as the input gene list file. The values shown in the pie chart are the percentage of genes classified to each GO term over the total number of genes in the list used.
