# Supplementary material for: Identification of putative regulatory regions and transcription factors associated with intramuscular fat content traits
Source: BMC Genomics. 2018 Jun 27;19:499. doi: 10.1186/s12864-018-4871-y (PMC6020320; doi:10.1186/s12864-018-4871-y)
Supplement: Supplementary file 16 — Over-represented TFBS motifs of: (A) EGR4 (NGFI-C aliases), (B) RUNX1 and (C) USF1 in genes into hotspot eQTL regions. (DOCX 240 kb) [file 12864_2018_4871_MOESM16_ESM.docx]

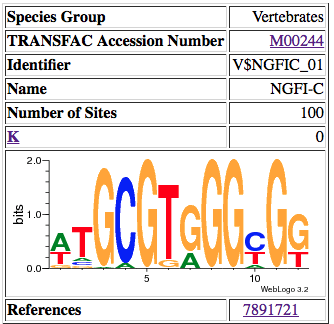

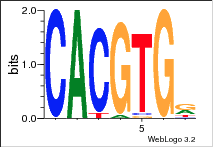

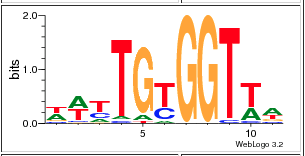


A

B

C

Additional file 16. Over-represented TFBS motifs of: (A) *EGR4* (*NGFI-C* aliases), (B) *RUNX1* and (C) *USF1* in genes into hotspot eQTL regions.
